# Reduction of myeloid‐derived suppressor cells in prostate cancer murine models and patients following white button mushroom treatment

**DOI:** 10.1002/ctm2.70048

**Published:** 2024-10-10

**Authors:** Xiaoqiang Wang, Shoubao Ma, Przemyslaw Twardowski, Clayton Lau, Yin S. Chan, Kelly Wong, Sai Xiao, Jinhui Wang, Xiwei Wu, Paul Frankel, Timothy G. Wilson, Timothy W Synold, Cary Presant, Tanya Dorff, Jianhua Yu, David Sadava, Shiuan Chen

**Affiliations:** ^1^ Department of Cancer Biology & Molecular Medicine Beckman Research Institute, City of Hope Duarte California USA; ^2^ Department of Hematology and Hematopoietic Cell Transplantation City of Hope Comprehensive Cancer Center Duarte California USA; ^3^ Department of Urology and Urologic Oncology Providence Saint John's Cancer Institute Santa Monica California USA; ^4^ Department of Surgery City of Hope Comprehensive Cancer Center Duarte California USA; ^5^ Integrative Genomics Core Beckman Research Institute, City of Hope Monrovia California USA; ^6^ Department of Computational and Quantitative Medicine, Beckman Research Institute, City of Hope Duarte California USA; ^7^ Department of Medical Oncology & Therapeutics Research City of Hope Comprehensive Cancer Center Duarte California USA

**Keywords:** clinical trial, myeloid‐derived suppressor cells (MDSCs), nutraceutical intervention, prostate cancer, single immune cell profiling, white button mushroom

## Abstract

**Background:**

In a previously reported Phase I trial, we observed therapy‐associated declines in circulating myeloid‐derived suppressor cells (MDSCs) with the administration of white button mushroom (WBM) tablets in prostate cancer (PCa) patients. These observations led us to hypothesise that WBM could mitigate PCa progression by suppressing MDSCs.

**Methods:**

We performed bidirectional translational research to examine the immunomodulatory effects of WBM consumption in both syngeneic murine PCa models and patients with PCa participating in an ongoing randomised Phase II trial (NCT04519879).

**Results:**

In murine models, WBM treatment significantly suppressed tumour growth with a reduction in both the number and function of MDSCs, which in turn promoted antitumour immune responses mediated by T cells and natural killer (NK) cells. In patients, after consumption of WBM tablets for 3 months, we observed a decline in circulating polymorphonuclear MDSCs (PMN‐MDSCs), along with an increase in cytotoxic CD8^+^ T and NK cells. Furthermore, single immune cell profiling of peripheral blood from WBM‐treated patients showed suppressed STAT3/IRF1 and TGFβ signalling in circulating PMN‐MDSCs. Subclusters of PMN‐MDSCs presented transcriptional profiles associated with responsiveness to fungi, neutrophil chemotaxis, leukocyte aggregation, and regulation of inflammatory response. Finally, in mouse models of PCa, we found that WBM consumption enhanced the anticancer activity of anti‐PD‐1 antibodies, indicating that WBM may be used as an adjuvant therapy with immune checkpoint inhibitors.

**Conclusion:**

Our results from PCa murine models and patients provide mechanistic insights into the immunomodulatory effects of WBM and provide a scientific foundation for WBM as a nutraceutical intervention to delay or prevent PCa progression.

**Highlights:**

- White button mushroom (WBM) treatment resulted in a reduction in pro‐tumoural MDSCs, notably polymorphonuclear MDSCs (PMN‐MDSCs), along with activation of anti‐tumoural T and NK cells.
- Human single immune cell gene expression profiling shed light on the molecular alterations induced by WBM, specifically on PMN‐MDSCs.
- A proof‐of‐concept study combining WBM with PD‐1 blockade in murine models revealed an additive effect on tumour regression and survival outcomes, highlighting the clinical relevance of WBM in cancer management.

## Graphical abstract

Our study involving both prostate cancer murine models and patients elucidates the cellular and molecular mechanisms by which white button mushroom nutraceutical intervention modulates the immune response in prostate cancer. It highlights the significant reduction of MDSCs as a key biological response to the treatment, thereby enhancing anti‐cancer immunity through the activation of cytotoxic T and NK cells.

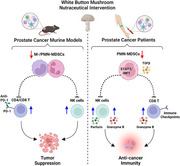

## 1 INTRODUCTION

Myeloid‐derived suppressor cells (MDSCs) are pathologically activated immature neutrophils (polymorphonuclear/PMN‐MDSCs) and immature monocytes (monocytic/M‐MDSCs) with potent immunosuppressive activity that proliferate and expand during cancer progression.^1^ These phenomena are notable in the progression of prostate cancer (PCa).^2^ MDSCs promote tumour progression by impairing the effectiveness of antitumour immunity provided by cytotoxic T cells and natural killer (NK) cells.^3^, ^4^ Therefore, reducing the number and function of MDSCs promotes anticancer immunity.^5^ Although there are some drugs being designed and developed in this area,^6^ nutraceutical interventions could be a useful approach given their minimal side effects.^7^ The idea of ‘food as medicine’ has been proposed to incorporate nutraceutical interventions into standardised clinical care and treatment protocols. However, there is often insufficient rigorous scientific evidence to validate such approaches.^8^, ^9^ For example, the utilisation of mushroom‐derived products as anticancer agents in cancer management is largely anecdotal in prior human experience. Most of this came from studies on non‐dietary medicinal mushrooms used in traditional medicine, rather than edible mushrooms used as food.^10^, ^11^

*Agaricus bisporus*, commonly known as the white button mushroom (WBM), is the most cultivated edible mushroom globally.^12^ It has been promoted as an anticancer food by both public and professional media.^13^ Despite these claims, the precise anti‐cancer mechanism of WBM remains undefined. We have conducted both preclinical^14^, ^15^, ^16^ and clinical^17^ studies to evaluate WBM as a nutraceutical intervention in prostate cancer. In our previous single‐arm Phase I clinical trial of WBM tablet consumption (NCT00779168), we confirmed that WBM tablets have a low‐toxicity profile and observed high participant compliance at the administered doses.^17^ Of particular significance, 13 of the 36 participants experienced a decrease in prostate‐specific antigen (PSA) levels upon WBM treatment, without affecting serum testosterone levels. We further observed therapy‐associated declines in circulating MDSCs (CD33^+^HLA‐DR^−^) in four patients with complete and partial responses.^17^ The decrease in PSA levels in response to therapeutic agents is typically caused by androgen receptor (AR) suppression and is associated with a delay in the onset of metastasis.^18^ Our subsequent reversed translational study with human PCa cell lines and patient‐derived xenograft (PDX) models in NOD scid gamma (NSG) mice enabled the identification of conjugated (9Z, 11E)‐linoleic acid (CLA‐9Z11E) as an AR antagonistic component of WBM, causing a decrease in PSA expression in human cancer cells and PDX tumours.^15^ Nevertheless, the mechanisms underlying the reduction in MDSCs observed after WBM treatment in responders among PCa patients remain unclear. The observed correlation between declining MDSCs and clinical responsiveness in PCa patients from the Phase I trial prompted us to design and conduct this two‐way translational study involving both murine models and patients.

In the current study, with the approval of WBM as an investigational new drug (IND) by the US FDA, we investigated the immune system responses to WBM consumption. We focused specifically on MDSCs by conducting experiments on murine models and analysing blood samples from PCa patients participating in our ongoing dual‐centre, two‐arm, open‐label randomised Phase II trial (NCT04519879). Our two‐way translational research strategy substantiates the immunomodulatory mechanism of WBM in affecting MDSCs, thereby reinforcing the foundation for considering WBM as a nutraceutical intervention for mitigating PCa progression.

## 2 RESULTS

### 2.1 WBM extract suppresses tumour growth both in prophylactic and therapeutic PCa mouse models

**FIGURE 1:**
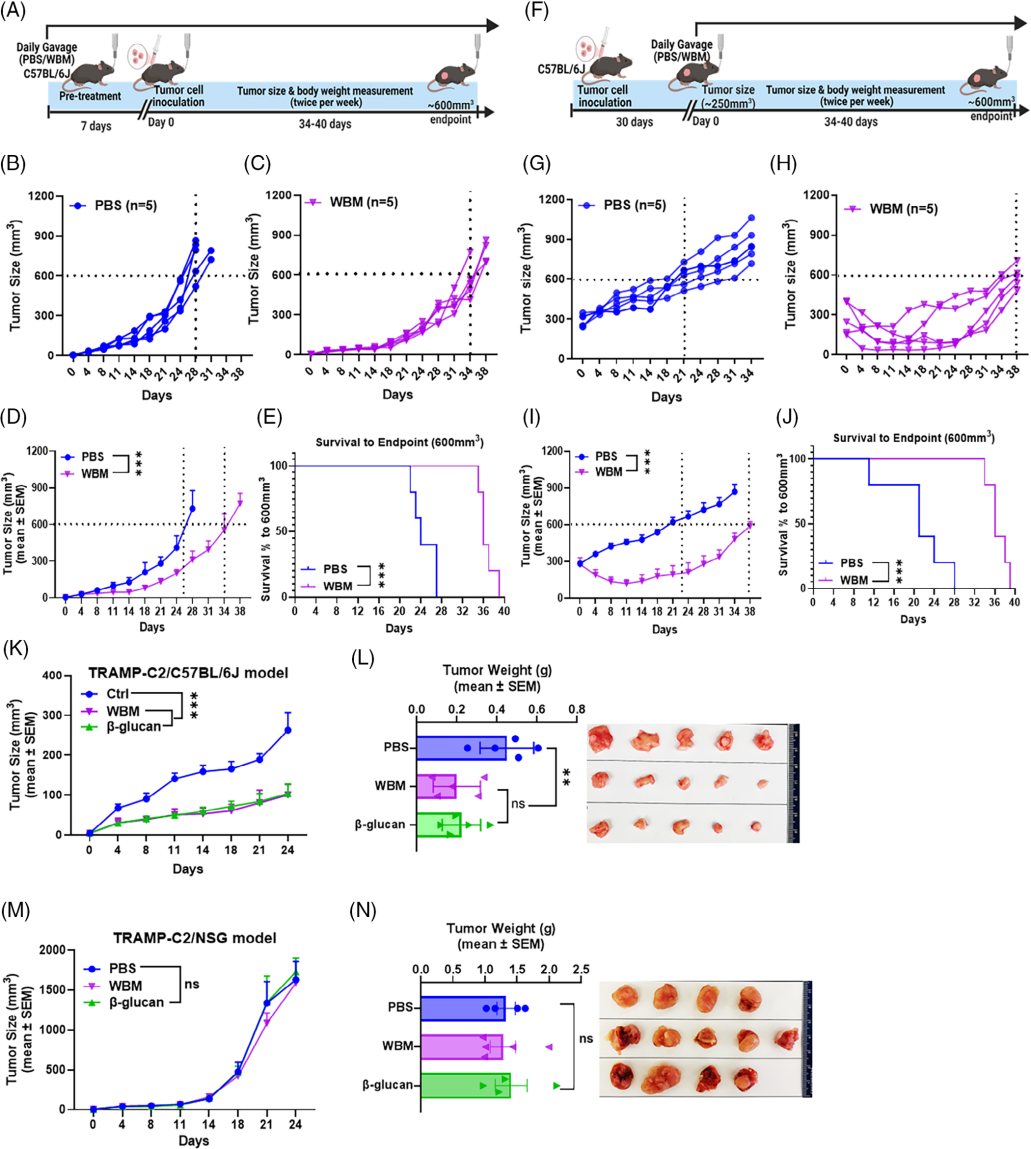
Effects of WBM extract on tumour growth in TRAMP‐C2 flank tumour xenograft models. (A) Schematic illustration of the prophylactic model of subcutaneous TRAMP‐C2 tumour xenografts in C57BL/6J mice treated with PBS or WBM extract (6 mg/mouse/day) in the indicated schedule. The (B, C) individual and (D) cumulative tumour growth curves post‐treatment application (PBS: *n* = 5; WBM: *n* = 5). (E) The Kaplan–Meier survival curves show the time it takes to reach a tumour volume of 600 mm^3^ in the prophylactic model. (F) Schematic illustration of the therapeutic model of subcutaneous TRAMP‐C2 tumour xenografts in C57BL/6J mice treated with PBS or WBM extract (6 mg/mouse/day) according to the specified timeline. The line graphs show (G, H) individual and (I) mean tumour growth rates following specified treatments (PBS: *n* = 5; WBM: *n* = 5). (J) The Kaplan–Meier survival curves show the time it takes to reach a tumour volume of 600 mm^3^ in the therapeutic model. (K) A composite tumour growth curve for subcutaneous TRAMP‐C2 tumour xenografts in C57BL/6J mice treated with PBS, WBM extract, or β‐glucan (PBS: *n* = 5; WBM: 6 mg/mouse/day, *n* = 5; β‐glucan: 1 mg/mouse/day, *n* = 5). (L) The bar graph demonstrates differences in tumour weight between the three treatment groups, which is accompanied by a photograph of the TRAMP‐C2 tumour xenografts in C57BL/6J mice. (M) A composite tumour growth curve for subcutaneous TRAMP‐C2 tumour xenografts in NSG mice, treated with PBS, WBM extract, or β‐glucan (PBS: *n* = 4; WBM: 6 mg/mouse/day, *n* = 5; β‐glucan: 1 mg/mouse/day, *n* = 4). (N) The bar graph demonstrates differences in tumour weight between the three treatment groups, which is accompanied by a photograph of the TRAMP‐C2 flank tumour xenografts in NSG mice. Data are presented as mean tumour volume ± SEM and were analysed using an ordinary one‐way ANOVA with a Tukey's post‐test for multiple comparisons. ns. non‐statistical significance, **p < .01, ***p < .001.

Prophylactic and therapeutic syngeneic mouse PCa models were established to demonstrate the antitumour activity of WBM extract. In the prophylactic model, C57BL/6J mice received a single daily dose of 6 mg/mouse of WBM extract in PBS via oral gavage for 7 days prior to tumour cell inoculation. After the pre‐treatment period, TRAMP‐C2 tumour cells (2 × 10^6^) were injected subcutaneously. The control group was given PBS only. Tumour measurements commenced on day 0 with the tumour cell inoculation (Figure 1A). Tumours in mice treated with PBS reached 600 mm^3^ by day 26, while those receiving WBM treatment required 34 days to reach the same size (Figure 1B and C). Administration of the WBM extract significantly delayed the growth of TRAMP‐C2 tumours and extended the survival of mice to the experimental endpoint (600 mm^3^ of tumours) (Figure 1D and E). These results suggest the potent antitumour activity of the WBM extract in prophylactic models. We then investigated the potential effectiveness of the WBM extract as a therapeutic agent. C57BL/6J mice were inoculated subcutaneously with 2 × 10^6^ TRAMP‐C2 cells. Once the tumours reached a size of 250 mm^3^, the tumour‐bearing mice were randomised into two groups: one receiving a single daily dose of 6 mg/mouse of WBM extract in PBS, and the other receiving only the PBS vehicle, for a period of 34–40 days. Tumour shrinkage measurements began on day 0 once the treatment commenced (Figure 1F). In this therapeutic model, tumours in mice treated with PBS reached 600 mm^3^ by day 21, whereas WBM extract treatment significantly reduced the tumour size at the onset of treatment and maintained the tumour size below 300 mm^3^ for 38 days (Figure 1G–I). Furthermore, treatment with WBM extract significantly prolonged survival until the experimental endpoint (Figure 1J). In summary, our findings suggest that WBM extract exhibits potent antitumour effects in both prophylactic and therapeutic PCa mouse models.

β‐Glucan from mushrooms has been extensively documented as an immune modulator.^19^ Therefore, we compared the antitumour effects of WBM extract and lentinan (purified β‐glucan from Shiitake mushrooms) in a prophylactic PCa model. The results showed that tumours in mice treated with either WBM extract or β‐glucan were smaller compared to those in PBS‐treated mice (Figure 1K and L). Similar results were obtained using a different syngeneic PCa mouse model (MyC‐CaP‐bearing FVB mice) (Figure S1A and B). Additionally, there were no noticeable changes in the body weight of C57BL/6J and FVB mice after the two treatments (Figure S1C and D), indicating that WBM extract and β‐glucan have limited systemic toxicity. To assess whether the WBM extract or β‐glucan exerted direct antitumour effects on cancer cells, an in vitro cell viability assay was conducted using the mouse PCa cell lines, TRAMP‐C2 and MyC‐CaP. Neither the WBM extract nor β‐glucan exhibited direct cytotoxicity against PCa cells (Figure S1E and F).

To determine whether the in vivo antitumour effects of the WBM extract or β‐glucan occur through immune modulation, we tested both treatments in TRAMP‐C2 bearing NOD scid gamma (NSG) mice, which are immunodeficient and lack mature T, B, and NK cells. Tumours in NSG mice treated with either WBM or β‐glucan grew as rapidly as those in PBS‐treated mice (Figure 1M and N). These results suggest that the antitumour efficacy of WBM extract and β‐glucan in PCa murine models would be contingent on the integrity of host immunity.

### 2.2 WBM extract treatment reduces the number and function of MDSCs in PCa mouse models

**FIGURE 2:**
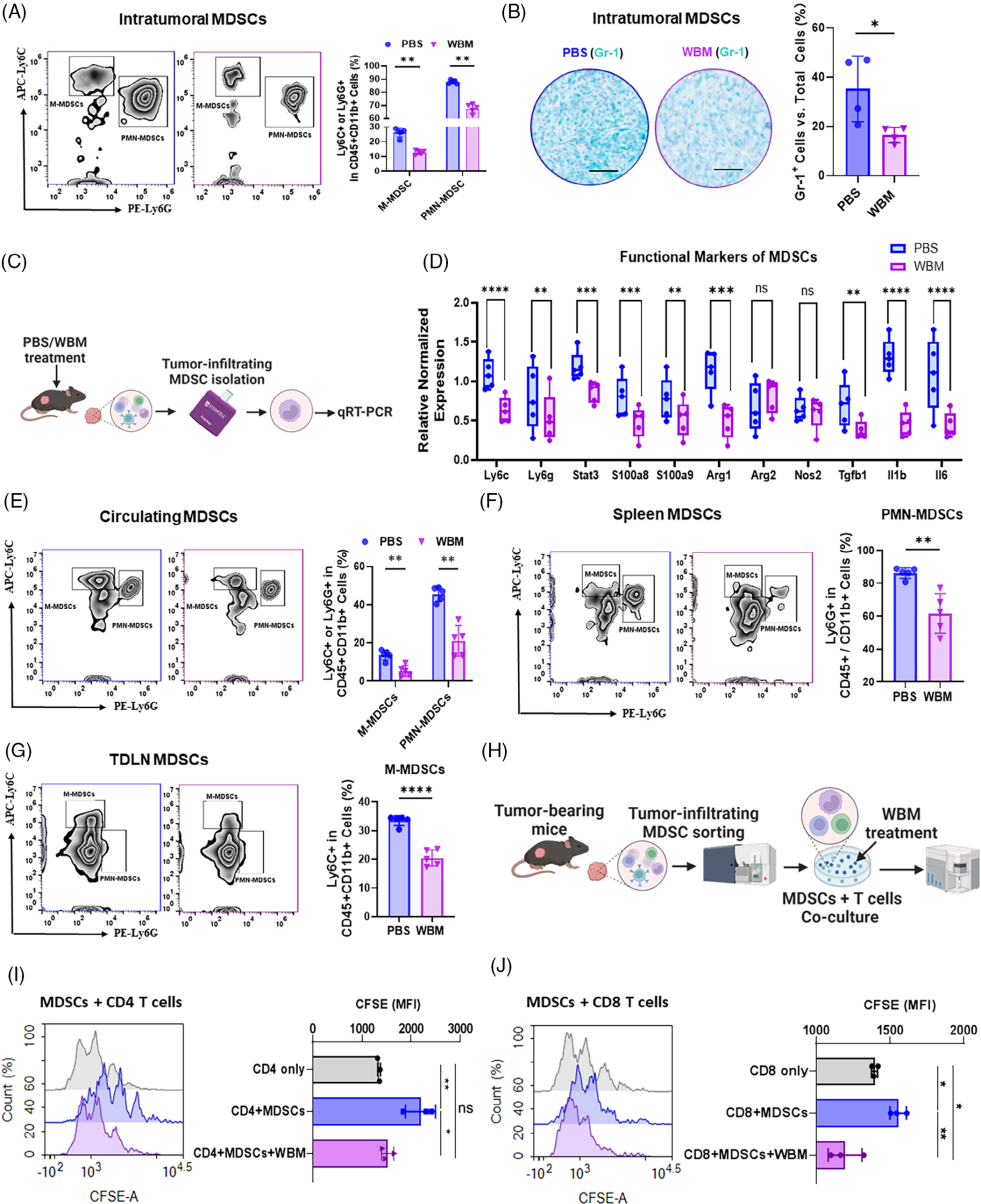
Impact of WBM extract on MDSC presence and activity in the TRAMP‐C2‐C57BL/6J mouse model. (A) Flow cytometry results show intratumoural M‐MDSCs (CD11b^+^/Ly6C^+^/Ly6G^−^) and PMN‐MDSCs (CD11b^+^/Ly6G^+^/Ly6C^−^) with respective quantitative comparisons (*n* = 5) between PBS‐ and WBM‐treated groups. (B) The representative IHC images (scale bar = 400 µm, *n* = 4) highlight Gr‐1 expression (Teal) in tumours, and the bar graph represents the corresponding quantitative analysis comparing PBS and WBM treatments. (C) The schematic diagram shows the process for isolating tumour‐infiltrating MDSCs from TRAMP‐C2 xenograft tumours in C57BL/6J mice treated with PBS or WBM extract for qRT‐PCR analysis. (D) The box plot compares the relative expression levels of key MDSC genes (*Ly6C*, *Ly6G*, *Stat3*, *S100a8*, *S100a9*, *Arg1*, *Arg2*, *Nos2*, *Tgfb*, *Il1b*, *Il6*) in tumour infiltrating MDSCs extracted from xenograft tumours treated with either PBS or WBM (*n* = 5). The flow cytometry results and corresponding quantitative comparisons of (E) circulating M‐MDSCs and PMN‐MDSCs, (F) spleen M‐MDSCs and PMN‐MDSCs, and (G) M‐MDSCs and PMN‐MDSCs in tumour‐draining lymph nodes (TDLNs) between PBS‐ and WBM‐ treated groups (*n* = 5). (H) Schematic illustration of tumour‐infiltrating MDSC isolation for MDSCs‐CD4/CD8 T cells co‐culture assay in the presence of WBM extract (10 µg/mL). The mean fluorescence intensity (MFI) of (I) CFSE‐positive CD4 T cells and (J) CFSE‐positive CD8 T cells was detected as an index of proliferative CD4/CD8 T cells. Statistical evaluations were performed using one‐way ANOVA with Tukey's post‐test for multiple comparisons. Significance notations: ns (non‐significant), *p < .05, **p < .01, ***p < .001, ****p < .0001.

In our earlier Phase I clinical trial, patients with biochemically recurrent PCa treated with WBM showed therapy‐associated decline in circulating MDSCs.^17^ Based on these observations in patients in Phase I trials, we initially assessed the changes in MDSCs after exposure to WBM in xenograft tumours, blood, spleen, and tumour‐draining lymph nodes (TDLNs) from immune‐competent mice using flow cytometry. We further assessed xenograft tumours through immunohistochemistry (IHC). Flow cytometry demonstrated that WBM extract treatment reduced the percentage of both intratumoural M‐MDSCs (CD11b^+^/Ly6C^+^/Ly6G^−^) and PMN‐MDSCs (CD11b^+^/Ly6G^+^/Ly6C^−^) (Figure 2A). IHC further confirmed the decrease in Gr‐1 positive MDSCs in WBM‐treated tumour tissues (Figures 2B and S2A). Next, we evaluated the expression of functional genes in tumour‐infiltrating MDSCs isolated from PBS‐ or WBM‐treated xenograft tumours (Figure 2C), including surface markers (*Ly6c, Ly6g*), cytokines (*S100a8*, *S100a9*, *Il1b*, *Il6*, *Tgfb*), immune suppressive enzymes (*Arg1*, *Arg2*, *Nos2*), and transcription factors (*Stat3*). The results demonstrated that the WBM extract suppressed the expression of MDSC signature genes such as *S100a8, S100a9, Arg1, Il1b, Il6, Tgfb* and *Stat3* (Figure 2D). Additionally, there was a reduction in the percentages of M‐MDSCs and/or PMN‐MDSCs in the blood, spleen, and TDLNs following WBM extract treatment in the mouse models (Figure 2E–G). These data suggest that WBM extract treatment reduces the number of intratumoural and peripheral MDSCs in PCa mouse models. To further evaluate the suppressive activity of intratumoural MDSCs on T cell proliferation and to assess whether WBM treatment could counteract these effects, MDSCs were sorted from xenograft tumours and CD4/CD8 T cells were sorted from spleens. These two cell types were co‐cultured at a ratio of 1:2 in the presence of WBM extract at 10 µg/mL. T cell proliferation was determined by measuring the mean fluorescence intensity (MFI) of carboxyfluorescein succinimidyl ester (CFSE)‐positive cells, which indicates their proliferative activity (Figure 2H). The results demonstrated that MDSCs co‐cultured with T cells suppress the proliferation of CD4^+^ T (Figure 2I) and CD8^+^ T (Figure 2J) cells, whereas the addition of WBM extract can restore T cell proliferation despite the presence of MDSCs (Figure 2I and J).

Previous reports have indicated that yeast‐derived β‐glucans suppress MDSCs by inducing apoptosis.^20^ To investigate this, MDSCs isolated from xenograft tumours in C57BL/6J mice were treated with two concentrations of WBM or β‐glucan at concentrations of 1 µg/mL and 10 µg/mL for 24 h, followed by flow cytometric analysis of apoptosis and Arg1 expression. The concentration of WBM extract or β‐glucan (1 µg/mL and 10 µg/mL) applied to MDSCs was optimised using human monocytic THP‐1 cells, an established cell line for screening the cytotoxic and immune modulation activities of mushroom products.^21^, ^22^ The results indicated that 10 µg/mL of WBM or β‐glucan were non‐cytotoxic and exhibited immune‐modulating activity by stimulating cytokine expression in THP‐1 cells (Figure S2B and C). The flow‐cytometry results on MDSCs treated with WBM extract or β‐glucan showed that high concentration (10 µg/mL) of β‐glucan and WBM extract significantly induced MDSC apoptosis (Figure S2D) and suppressed Arg1 expression in MDSCs (Figure S2E). Taken together with the above results, these findings suggest that WBM extract treatment reduces the number of both intratumoural and peripheral MDSCs in PCa mouse models by inducing apoptosis and inhibiting their immunosuppressive functions against T cells.

### 2.3 WBM extract facilitates T‐cell‐mediated immune response in PCa mouse models

**FIGURE 3:**
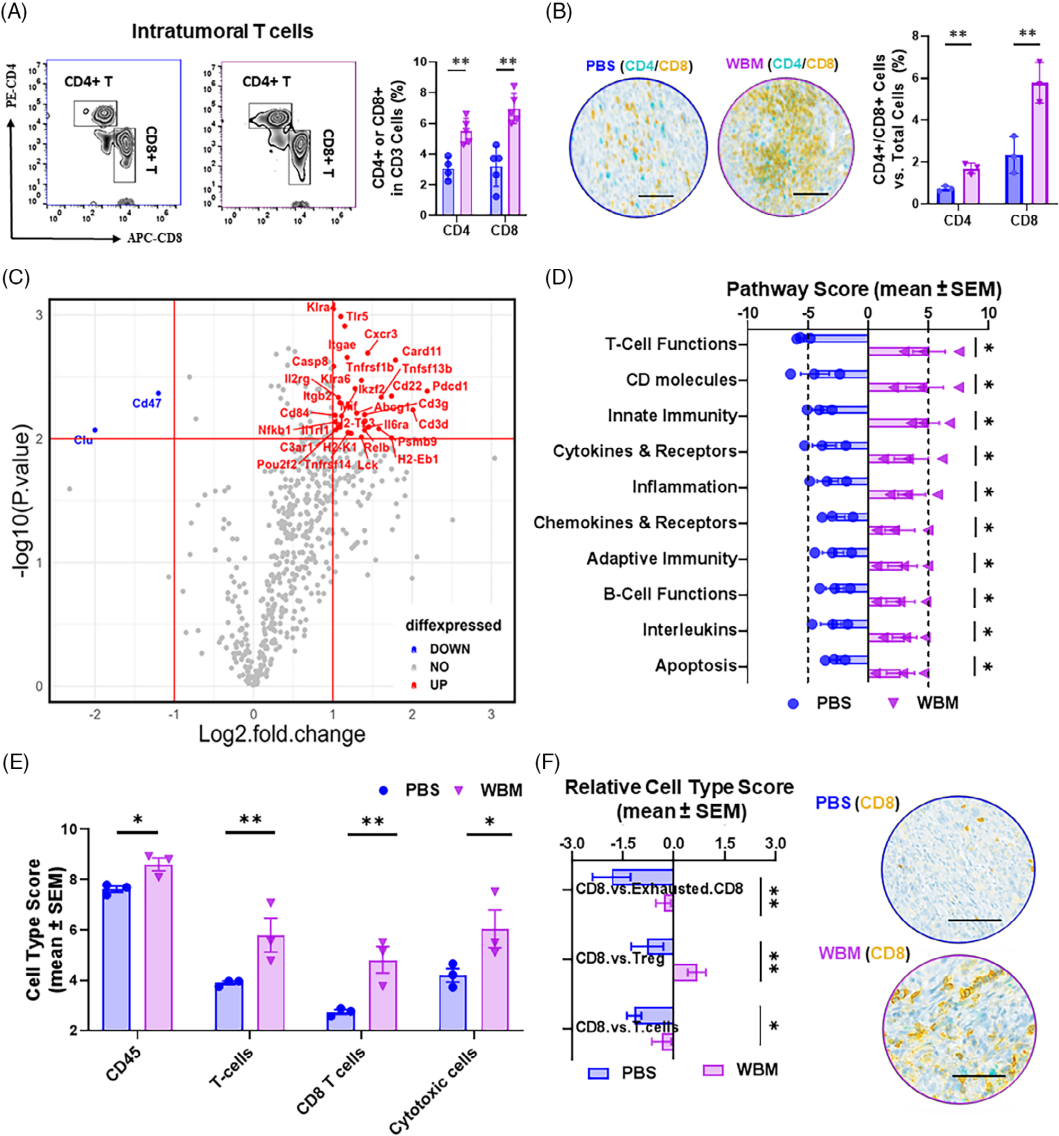
WBM extract promotes T‐cell‐mediated response in TRAMP‐C2 flank tumour xenografts in C57BL/6J mice. (A) The flow cytometry results of intratumoural CD4^+^ T cells (CD3^+^/CD4^+^) and CD8^+^ T cells (CD3^+^/CD8^+^) with accompanying quantitative analysis comparing PBS‐ and WBM‐treated (*n* = 5) groups. (B) Images from multiplex IHC staining (scale bar = 400 µm) for CD4^+^ T cells (Teal) and CD8^+^ T cells (Yellow) in tumours, with quantitative comparisons between PBS‐ and WBM‐treated (*n* = 3) groups. (C) The volcano plot shows differential gene expression in tumours (*n* = 3), distinguishing between PBS and WBM treatments with the nCounter^®^ PanCancer IO 360™ Panel. The plot marks non‐significant genes as gray dots, down‐regulated genes as blue dots (*n* = 2, log2 FC < –1, –log10 *P* > 1), and upregulated genes as red dots (*n* = 31, log2 FC > 1, –log10 *P* > 1). (D) The pairwise box plots showcase PanCancer IO 360 pathway scores enriched in PBS‐ and WBM‐treated xenograft tumours (*n* = 5), which were calculated by nSolver 4.0 software with pathway score algorithm. (E) The bar graph shows immune cell scores enriched in tumours for PBS‐ and WBM‐treated groups (*n* = 3), which were derived using the nSolver 4.0 software. (F) The box plots represent relative CD8^+^ T cell scores to other types of T cells and corresponding IHC images (scale bar = 400 µm, *n* = 3) for CD8^+^ T cells (Yellow), showcasing the differences between PBS‐ and WBM‐treated tumours. Data are presented as mean ± SEM and analysed using ordinary one‐way ANOVA with a Tukey's post‐test for multiple comparisons. Notations indicate statistical significance as follows: ns (non‐significant), *p < .05, **p < .01, ***p < .001, ****p < .0001.

MDSCs are known for their immunosuppressive activity and their ability to inhibit T cell function.^23^ In the experiments mentioned above, we observed that WBM treatment led to a reduction in both the number and function of MDSCs. Next, we examined the consequential effects on the function of intratumoural T cells and their capability of tumour infiltration. First, we examined the ratio of tumour infiltrating CD4/CD8^+^ T cells in PCa models using flow cytometry and multiplex IHC. Flow cytometry revealed an increase in intratumoural CD4^+^ and CD8^+^ T cells in mice treated with the WBM extract (Figure 3A and B). We also investigated the spatial intratumoural immune infiltration patterns (topography) of CD4^+^ and CD8^+^ T cells using multiplex IHC, which demonstrated a pronounced accumulation of CD4^+^ and CD8^+^ T cells in the core of WBM‐treated tumours (Figure S3A). In comparison, most CD4/CD8^+^ T cells were concentrated at the tumour boundaries, with fewer cells infiltrating the tumour bed in PBS‐treated tumours (Figure S3A). Additionally, white blood cell differential evaluation of PCa mouse models suggested a decrease in total neutrophils and an increase in lymphocytes (Figure S3B). Elevated levels of CD4/CD8^+^ T cells were also observed in the blood, spleen, and TDLNs of WBM‐treated mice by flow cytometry (Figure S3C–E).

To gain further insight into the effect of WBM consumption on the tumour immune response, murine xenograft tumours were analysed using the nCounter PanCancer IO 360 Gene Expression Panel to profile the expression of 770 genes related to the tumour microenvironment and immune response.^24^ Among these genes, *Cd47* and *Clu* were downregulated, whereas 31 genes were upregulated in WBM‐treated tumours compared with those in PBS‐treated controls (Figure 3C). CD47 inhibits phagocytosis by macrophages, thereby enabling tumour cells to evade immune surveillance.^25^ Among the upregulated genes, *Pdcd1* (encoding PD‐1, a checkpoint inhibitor that prevents T cells from attacking tumours^26^), *Cxcr3* and *Cxcl13* (associated with T cell trafficking and function^27^), *Tnfsf13b* (encoding BAFF) and *Tnfsf4* (encoding OX40L) (T cell co‐stimulating factors^28^), and *Card11* are important for antigen‐induced lymphocyte activation^29^ (Figure 3C and Table S1). Pathway enrichment analysis revealed a positive enrichment of upregulated genes in pathways associated with ‘T cell functions’, ‘CD molecular’, ‘innate immunity’, ‘cytokines & receptors’, and ‘inflammation’ (Figure 3D and Table S2). Furthermore, NanoString analyses generated cell type scores that assessed the abundance of cells based on cell type‐specific gene expression,^30^ indicating that WBM‐treated tumours displayed a higher abundance of CD45^+^ cells, CD8^+^ T cells, and cytotoxic cells (Figure 3E). In addition to the cell type score, the Relative Cell Type Score was determined by comparing the CD8 abundance relative to exhausted CD8^+^ T cells, Treg cells, and overall T cell abundance. These results further support the finding that CD8^+^ T cells increased relative to other cell types following WBM treatment (Figure 3F). Taken together, these findings suggest that WBM treatment spatially and molecularly enhances T cell infiltration into tumours and promotes antitumour activity.

In addition to the observed alterations in T cells, the NanoString cell‐type algorithm also computed abundance scores for various other cell types, including dendritic cells (DCs), macrophages, Treg cells, and NK cells in xenograft tumours. The cell‐type algorithm suggested an increase in NK cells in WBM‐treated tumours without alterations in other cell types (Figure S4A). Flow cytometry was used to further characterise the tumour‐infiltrating cell types. These results showed that WBM treatment did not induce changes in the number of DCs (Figure S4B), macrophages (Figure S4C), and Treg cells (Figure S4D). However, the number of intratumoural NK cells increased after WBM treatment (Figure S4E).

### 2.4 WBM consumption by PCa patients reduces circulating PMN‐MDSCs accompanied by activation of T and NK cells

**FIGURE 4:**
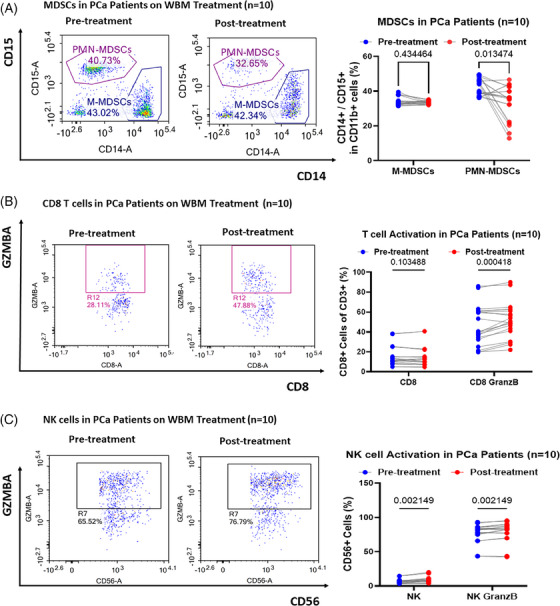
Impact of WBM consumption on circulating MDSCs, T, and NK cell dynamics in prostate cancer (PCa) patients. Flow cytometry results show the level of (A) circulating M‐MDSCs (CD11b^+^/CD14^+^) and PMN‐MDSCs (CD11b^+^/CD15^+^), (B) active CD8^+^ T cells (CD8^+^/Granzyme B^+^), and (C) active NK cells (CD56^+^/Granzyme B^+^) in PCa patients’ whole blood samples (*n* = 20, paired samples from 10 patients) collected at timepoint of pre‐treatment (first days of enrolment) and post‐treatment (3 months post‐WBM treatment). Each subfigure has a corresponding pairwise line‐point scatter plot, quantifying cell counts in patient blood samples taken pre‐ and post‐treatment. Data are presented using pairwise line‐spot plots and analysed using ordinary one‐way ANOVA with Tukey's post‐test for multiple comparisons. Significance levels are noted as ns (non‐significant), *p < .05, **p < .01, ***p < .001, ****p < .0001.

Building on insights gained from mouse models and Phase I trials in PCa patients, we investigated whether the consumption of WBM induces changes in the identified cell types, particularly MDSCs, T cells, and NK cells, in PCa patients participating in our ongoing randomised Phase II trial. To accomplish this, we identified 10 PCa patients undergoing active surveillance for enrolment in the WBM treatment group. Additionally, 8 PCa patients were identified in the control group without WBM treatment. Whole blood samples were collected at baseline and after 3 months of WBM treatment. Subsequently, the 18 pairs of whole blood samples were processed for multiplex flow cytometry. M‐MDSCs were defined as CD33^+^, HLA‐DR^–^, CD14^+^ and CD15^–^, whereas PMN‐MDSCs were defined as CD33^+^, HLA‐DR^–^, CD14^–^, and CD15^+^. The analysis revealed a significant reduction in PMN‐MDSCs, with minimal changes in M‐MDSCs, among patients (*n* = 10) who received WBM for 3 months (Figure 4A). However, no significant differences were observed in MDSCs in the untreated control patients (*n* = 8) (Figure S5). The consistent changes in PMN‐MDSCs observed in both animal models and patients suggest that PMN‐MDSCs are preferentially responsive to WBM treatment.

We then investigated whether a reduction in MDSCs leads to T cell activation in these patients. A T cell activation panel including antibodies against CD3, CD4, CD8, TNFα, IFN‐γ, and granzyme B was designed to assess the impact of WBM treatment on CD4 and CD8 T cells. These results demonstrated that WBM treatment induced CD8^+^ T cell activation by promoting Granzyme B production (Figure 4B). No significant changes were observed in the total number of CD4^+^ and CD8^+^ T cells (Figure S6A and B) or the number of TNF‐α‐ or IFN‐γ‐positive CD4^+^ and CD8^+^ T cells (Figure S6C and D) after the WBM treatment. In addition, we evaluated the effects of WBM on T cell exhaustion. We also examined the expression of surface immune checkpoint proteins (PD‐1, CTLA‐4, and TIM3) that downregulate T cell activation to maintain peripheral tolerance.^31^ Notably, WBM treatment increased the proportion of PD‐1 and CTLA4 double‐positive CD4^+^ T cells (Figure S7).

Finally, we also examined the activation and exhaustion of NK cells in PCa patients treated with WBM using flow cytometry and a panel of antibodies against CD56, TNFα, IFN‐γ, Granzyme B, TIGIT, and NKG2A. The results showed that both total NK cells and Granzyme B positive NK cells increased with WBM treatment (Figure 4C), while the number of TNFα‐ or IFN‐γ‐positive NK cells remained comparable to pre‐treatment samples (Figure S8A and B), whereas WBM treatment did not induce changes in the number of TIGIT and NKG2A double‐positive NK cells (Figure S8A and C). The observed changes in T and NK cells in PCa patients were consistent with the results from the animal models, suggesting the translatability of WBM bioactivity to selective immune cells in preclinical mouse models.

### 2.5 WBM intake by PCa patients modulates PMN‐MDSCs, T, and NK cells’ functions at the single‐cell level

**FIGURE 5:**
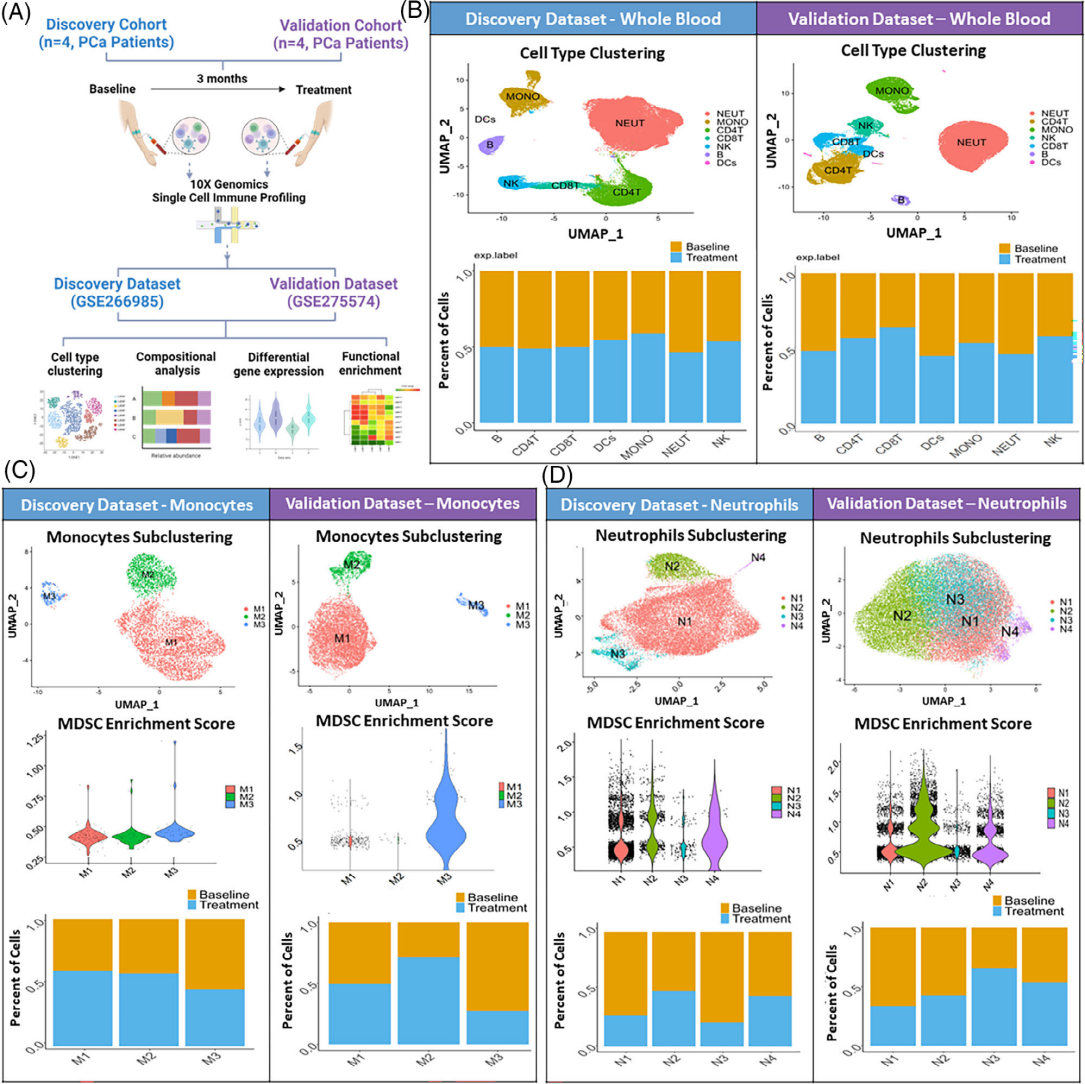
Effects of WBM intake on circulating immune cell profile in prostate cancer (PCa) patients at the single‐cell level. (A) Schematic diagram depicts how single immune cell gene expression profiling was conducted, which involved a discovery cohort (*n* = 4 patients) and a validation cohort (*n* = 4 patients). Paired whole blood samples were collected from patients in each cohort of ‘Baseline’ (first day of enrolment) and ‘Treatment’ (3 months of WBM treatment). They were independently processed for single‐cell RNA sequencing. The discovery dataset (GSE266985) included 4 paired samples (*n* = 8) from the discovery cohort. The validation dataset (GSE275574) included 4 paired samples (*n* = 8) from the validation cohort. Both datasets were used for cell type clustering, compositional analysis, differential gene expression analysis, and functional enrichment analysis. (B) Seurat analysis of single‐cell RNA sequencing data (Discovery vs. Validation) identified various immune cell populations (neutrophils, monocytes, CD4^+^ T cells, CD8^+^ T cells, NK cells, B cells, and dendritic cells (DCs)) in whole blood samples of ‘Discovery’ and ‘Validation’ Datasets. The bar charts for each dataset show the proportions of major immune cell types in Baseline versus Treatment samples. The proportions were determined by dividing the number of specific cell types from each group (Baseline vs. Treatment) by the total number of cells within the corresponding cluster. (C) Unbiased Seurat clustering of monocytes are depicted in a UMAP, identifying 3 distinct monocyte clusters (M1–M3) in the discovery and validation datasets. The violin plots display the relative MDSC scores sorted by monocyte subclusters. The bar charts show the proportions of cells of each monocyte subcluster contributed from ‘Baseline’ and ‘Treatment’ samples. (D) Neutrophil‐specific Seurat clustering analysis is depicted in a UMAP, identifying 4 distinct neutrophil clusters (N1–N4) in both datasets. The violin plots display the relative MDSC scores for neutrophil subclusters. The bar charts show an overview of changes in neutrophil cluster proportions in samples from baseline versus treatment.

Following our observations of the selective effects of WBM consumption on MDSCs, T cells, and NK cells in PCa murine models and patients, we next examined whether WBM consumption modulates the profile and transcriptional landscape of additional circulating immune cells in PCa patients. A non‐biased single immune cell profiling analysis was applied to eight pairs of whole blood samples collected from eight PCa patients who were under active surveillance, with samples collected before and after 3 months of WBM treatment. As shown in Figure 5A and Table S3, the discovery dataset (GSE266985) was derived from a discovery cohort of four patients, comprising a total of 18 982 cells from baseline samples (with individual contributions of 3077, 1925, 8441, and 5539 cells) and 12 668 cells from samples taken after 3 months of treatment (3754, 2652, 3617, and 2645 cells). The validation dataset (GSE275574) was created from a validation cohort of another four patients, totalling 35 378 cells from baseline (8805, 4642, 9541, and 12 390) and 37 883 cells from samples taken after 3 months of treatment (11 875, 7281, 8415, and 10 312). Cell type clustering and annotation assays revealed the major cell types that were proportionally present in the blood, including neutrophils, monocytes, CD4^+^ T cells, CD8^+^ T cells, NK cells, B cells, and DCs in both discovery and validation datasets (Figures 5B and S9A). Among the major cell types, neutrophils, monocytes, CD4^+^ T cells, CD8^+^ T cells, and NK cells were treatment‐responsive, showing changes in cell composition when comparing the per cent distribution of cells from baseline and 3‐month samples in both the discovery and validation datasets, whereas the composition of other cell types remained unchanged after treatment (Figure 5B).

PMN‐MDSCs are pathologically activated immature neutrophils, and M‐MDSCs are derived from immature monocytes.^32^ To annotate and elucidate the specific response of MDSCs within the monocytes and neutrophils, we performed subclustering analyses on monocytes and neutrophils individually and assigned MDSCs enrichment scores to subclusters based on previously described methods.^33^ There were three subclusters of monocytes (M1–M3) in both discovery and validation datasets (Figures 5C and S9B). Among these, subcluster M3 demonstrated the highest MDSC scores and showed a trend of decrease in the percentage of cells after WBM treatment (Figure 5C). Four subclusters (N1–N4) were identified within neutrophils (Figures 5D and S9C). These four subclusters exhibited varying degrees of MDSC enrichment scores (Figure 5D). In the discovery dataset, the percentages of subclusters N1 and N3 decreased significantly following WBM treatment. Similarly, in the validation dataset, the percentages of subcluster N1 and N2 showed a notable decrease after WBM treatment (Figure 5D).

**FIGURE 6:**
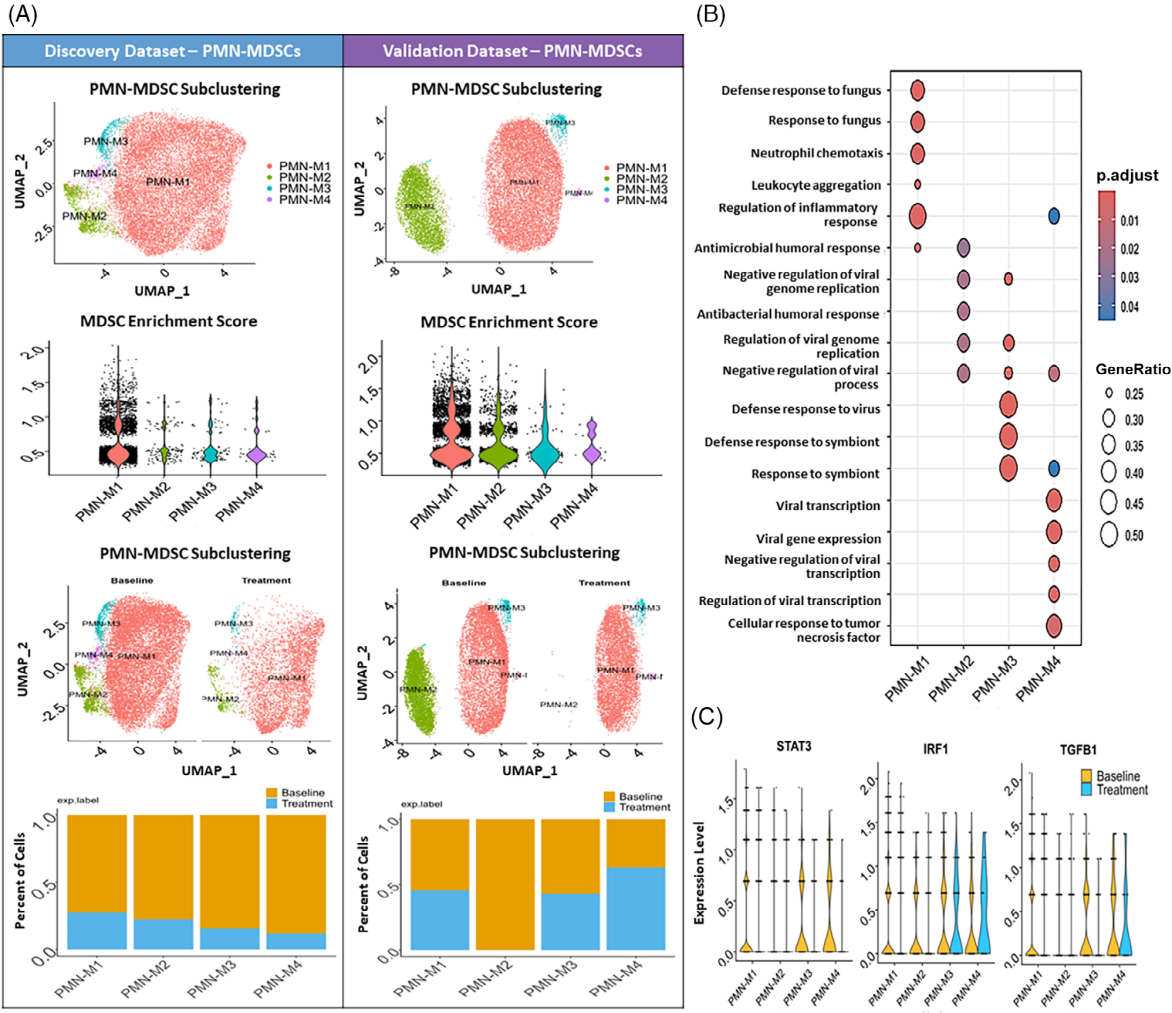
Impact of WBM intake on PMN‐MDSCs numbers and function in prostate cancer patients at the single‐cell level. (A) Unbiased Seurat clustering analysis of putative PMN‐MDSCs in a UMAP, identifying four distinct subclusters (PMN‐M1–M4) in both the discovery and validation dataset. The violin plots demonstrate the relative MDSC scores across the four putative PMN‐MDSC clusters in each dataset, showing similar levels of MDSC gene signature expression. The feature plot and bar chart show the changes in each putative PMN‐MDSC cluster post‐treatment, indicating a reduction across all four clusters in discovery dataset and one cluster in validation datasets. (B) The bubble plot represents Gene Ontology Biological Process (GO‐BP) gene signature scores for the PMN‐MDSC clusters from discovery dataset. The bubble shading indicates *p*‐value significance, and the bubble size corresponds to the number of genes associated with each process. (C) The violin plots show the expression levels of *STAT3*, *IRF1* and *TGFB1* within each PMN‐MDSC cluster from discovery dataset, providing insights into the gene expression within these cells.

To gain a deeper understanding of the molecular characteristics of PMN‐MDSCs after WBM treatment, subclusters N0 and N3 of neutrophils in discovery dataset and subclusters N1 and N2 in the validation dataset, which were enriched as putative PMN‐MDSCs, were subjected to additional clustering and functional enrichment analyses. Four distinct subclusters (PMN‐M1–M4) were identified in putative PMN‐MDSCs from both the discovery and validation datasets (Figures 6A and S10A). In the discovery dataset, the subcluster PMN‐M1 showed a relatively high MDSC enrichment score. The UMAP and composition clearly demonstrated a significant decrease in both the number and percentage of the four putative PMN‐MDSC subclusters after WBM treatment. Similarly, in the validation dataset, the subcluster PMN‐M2 exhibited a relatively high MDSC enrichment score, and the UMAP and composition further revealed a clear reduction in both the number and percentage of subcluster PMN‐M2 following WBM treatment (Figure 6A). Subsequently, we conducted functional enrichment analysis for each subcluster in the discovery dataset. The predominant function of subcluster PMN‐M1 in the discovery dataset was associated with ‘defence response to fungus’, ‘response to fungus’, ‘neutrophil chemotaxis’, and ‘leukocyte aggregation’, while subclusters PMN‐M2 to M4 were implicated in the negative regulation of the immune response (Figure 6B). Previous studies have indicated that neutrophilic MDSCs constitute a unique subset that responds to pathogenic fungi.^34^ Our findings align with the existing literature, indicating that the subcluster of putative PMN‐MDSCs was the primary cell type responding to WBM treatment in ‘response to the fungus.’ We further investigated the signature genes that regulate MDSCs differentiation and immunosuppressive functions, including *ARG1, PTGS2, CXCR2, TGFB1, IL1b, MMP9, STAT3*, and *IRF1*.^1^, ^35^ The results showed that WBM treatment suppressed the expression of *TGFB1*, *STAT3*, and *IRF1* in PMN‐MDSCs (Figure 6C) without notable changes in other genes (Figure S10B). STAT3 and IRF1 are key transcriptional regulators of MDSC expansion and survival.^36^, ^37^ Therefore, WBM treatment, leading to the downregulation of *STAT3* and *IRF1* in PMN‐MDSCs, may impair their immunosuppressive functions.

Flow cytometry analyses from both the murine PCa model and PCa patients treated with WBM showed an increase in T and NK cell populations. Consequently, we also examined the expression of T cell activation‐ and exhaustion‐related genes, including *PDCD1*, *CTLA4, HAVCR2, LAG3, TIGHT, IFNG, TNF*, and *GZMB* in T cells. Both the discovery and validation datasets from patient samples on T cells indicated that WBM treatment might alter the expression of *LAG3*, *GZMB, and IFNG* in CD8^+^ T cells (Figure S11A). This finding was consistent with our observations in patients’ whole blood samples analysed by flow cytometry (Figure 4B). We also examined the expression of genes associated with NK cell function, including *TIGIT*, *KLRC1*, *GZMB*, *PRF1*, *TNF*, and *IFNG*. Both the discovery and validation datasets showed that WBM treatment upregulated the expression of *GZMB* and *PRF1* expression in either NK proliferative cells or NK CD56 bright cells (Figure S11B). This was also in agreement with flow cytometry results from whole blood of patients treated with WBM (Figure 4C). In summary, unbiased single‐cell profiling analysis revealed consistent findings between the PCa murine model and patients, demonstrating that WBM treatment alters the number and function of PMN‐MDSCs, which subsequently enhances T and NK cell activity.

### 2.6 WBM treatment in combination with PD‐1 blockade delayed tumour growth in murine PCa models

**FIGURE 7:**
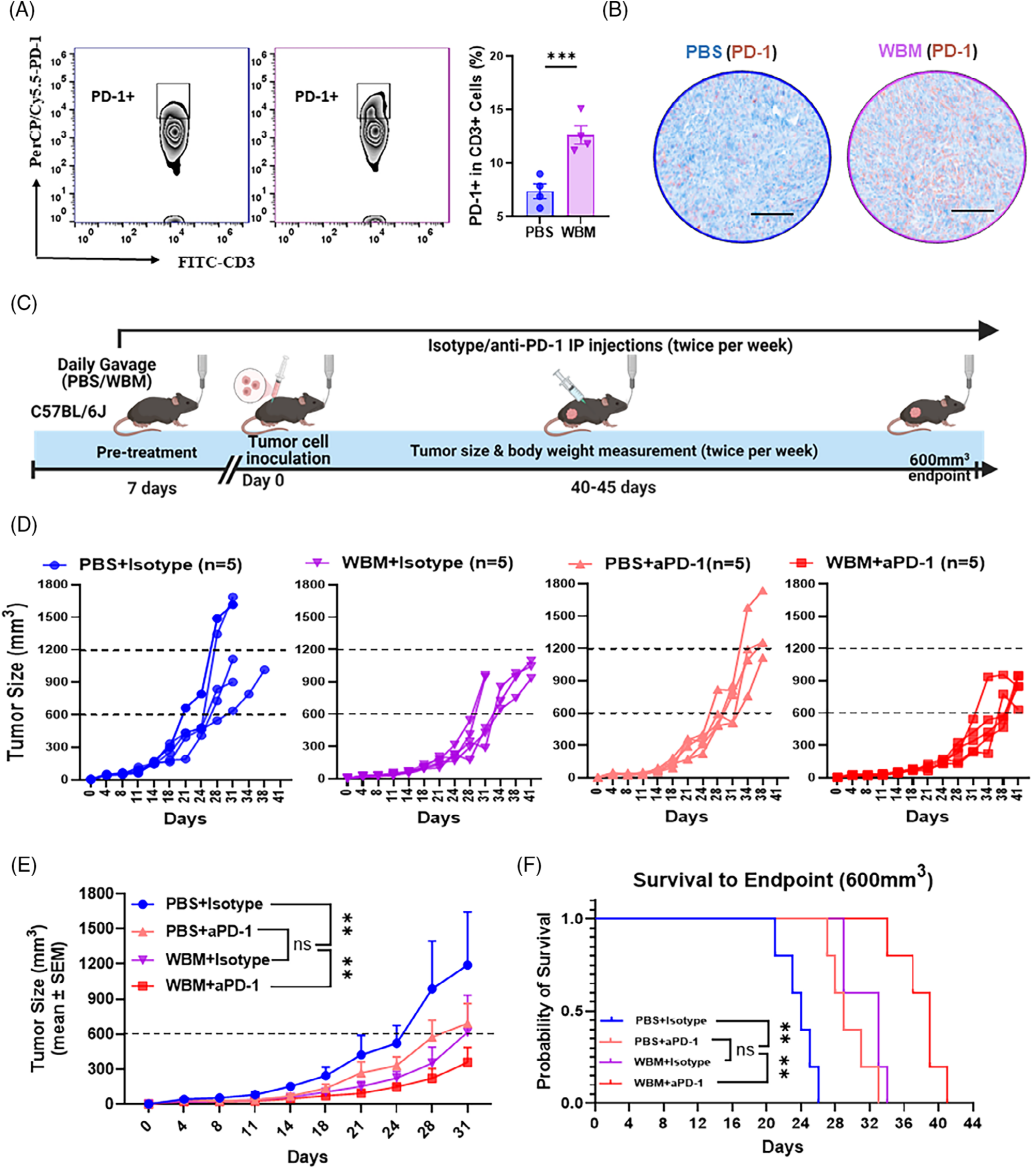
Combined WBM treatment and PD‐1 blockade slow tumour growth in TRAMP‐C2 flank tumour xenografts in C57BL/6J mice. (A) Flow cytometry results show that there are intratumoural PD‐1 positive CD3^+^ T cells in TRAMP‐C2 flank tumour xenografts in C57BL/6J mice, with quantitative comparisons (*n* = 4) between PBS‐ and WBM‐treated groups. (B). IHC staining images (scale bar = 400 µm, *n* = 4) display PD‐1 expression (Brown) in tumours from PBS‐ and WBM‐treated mice. (C) A schematic representation of the combination treatment protocol for subcutaneous TRAMP‐C2 flank tumour xenografts in C57BL/6J mice, treated with either PBS or WBM (6 mg/mouse/day) extract and either isotype or anti‐PD‐1 (aPD‐1) antibody (50 µg/mouse) via intraperitoneal (IP) injection. The line graphs show individual (D) and average (E) tumour growth dynamics for each treatment group (PBS+Isotype: *n* = 5; WBM+Isotype: *n* = 5; PBS+anti‐PD‐1: *n* = 5; WBM+anti‐PD‐1: *n* = 5). (F) The Kaplan–Meier survival curves show the time it took to reach a tumour volume of 600 mm^3^ for the four treatment groups within the therapeutic model. Data are presented as mean ± SEM and analysed using ordinary one‐way ANOVA with a Tukey's post‐test for multiple comparisons. Notations indicate statistical significance as follows: ns (non‐significant), *p < .05, **p < .01, ***p < .001, ****p < .0001.

Flow cytometry and IHC analyses of TRAMP‐C2 xenograft tumours indicated that WBM treatment enhanced the infiltration of PD‐1^+^ T cells (Figure 7A and B). Based on these observations, we conducted a proof‐of‐concept study that combined anti‐PD‐1 antibodies with WBM treatment. We hypothesised that blocking PD‐1 signalling may have an additive or synergistic antitumour effect when combined with WBM. We used the same TRAMP‐C2 xenograft model in C57BL/6J mice by intraperitoneal administration of anti‐PD‐1 monoclonal antibodies twice per week (Figure 7C) for 48–50 days. The results demonstrated a significant enhancement in tumour growth inhibition by WBM combined with PD‐1 blockade compared with monotherapy (Figure 7D and E). Additionally, the combination of WBM and anti‐PD‐1 mAbs prolonged the survival time to 48 days, whereas mice receiving monotherapy reached the endpoint by 40 days (Figure 7F). Next, we conducted a Bliss Independence calculation^38^, ^39^ to define the drug combination between WBM and anti‐PD‐1. The calculated combinatory effect (30.17%) was similar to the observed combinatory effect (30.1%), suggesting an additive effect (Figure S12). In summary, proof‐of‐concept studies suggest that there is a potential benefit of combining WBM with PD‐1 therapy to enhance PCa responsiveness to immune checkpoint inhibitors.

## 3 DISCUSSION

Prostate cancer (PCa) is classified as an immunologically cold tumour, marked by an immunosuppressive tumour microenvironment, partially due to the accumulation of intratumoural MDSCs.^40^ MDSCs are associated with restricted T/NK cell infiltration and, as a result, enhanced tumour progression.^2^, ^41^ In patients with PCa, elevated levels of PMN‐MDSCs have been reported in both the peripheral blood and tumour tissues, and these levels are correlated with tumour progression and poor prognosis.^42^, ^43^, ^44^, ^45^ MDSCs exert immunosuppression through various mechanisms, including but not limited to the generation of reactive oxygen and nitrogen species (ROS and RNS), as well as depletion of essential amino acids crucial for T cell function, which is mediated by the expression of arginase (Arg) and indoleamine 2,3‐dioxygenase (IDO).^2^ Several established chemotherapeutic drugs appear to decrease the accumulation and function of MDSCs, while others are under investigation.^5^, ^6^ However, the results from previous studies in both animal models and limited human trials have been inconsistent for various cancers, including PCa.^46^

**FIGURE 8:**
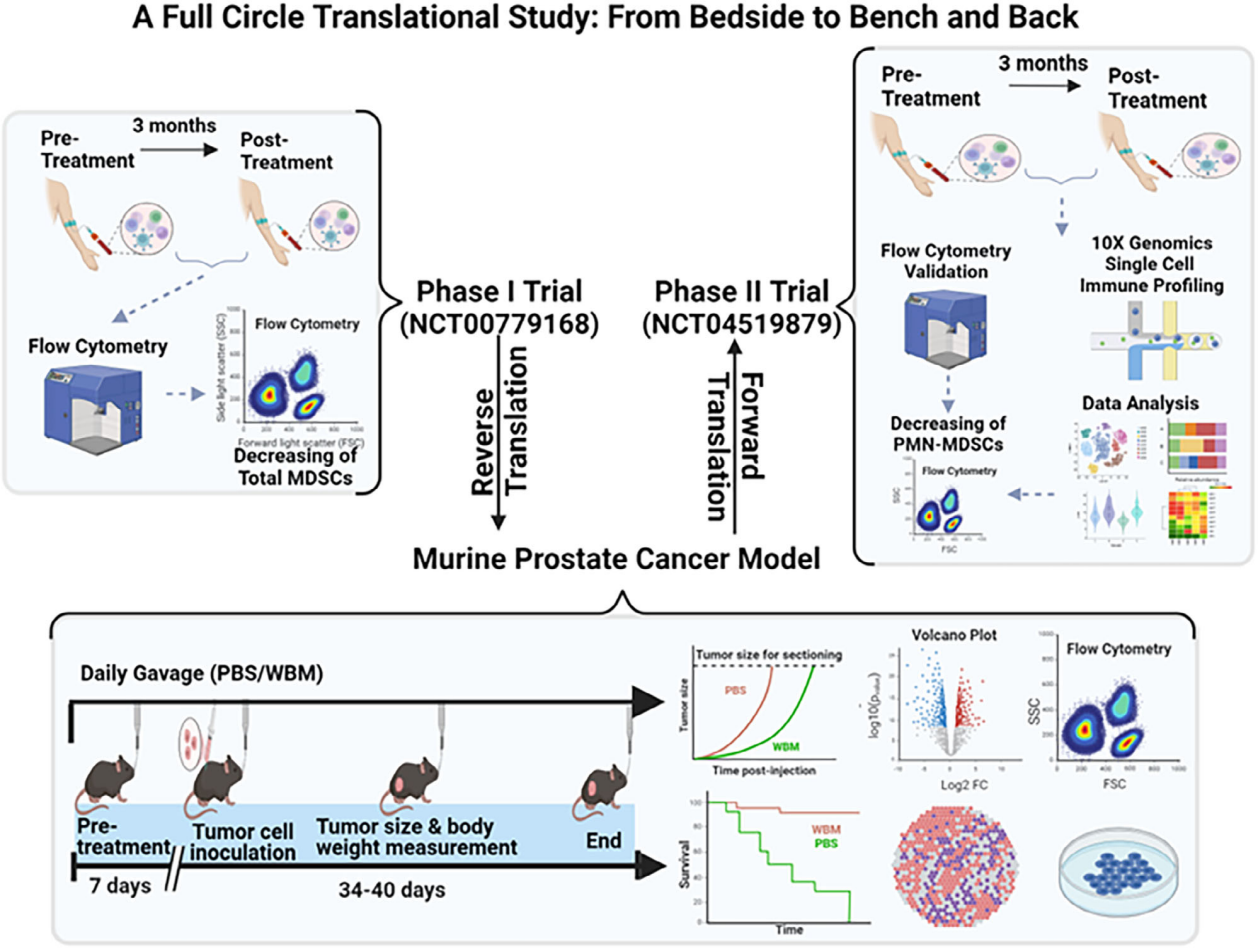
A Full circle translational research design to explore the immunoregulatory effects of WBM in PCa patients. The initial single‐arm Phase I clinical trial involving WBM tablet consumption (NCT00779168) in PCa patients identified circulating MDSCs as immune cells responsive to WBM treatment. Building upon these findings, the current study includes experiments on PCa murine models and analysis of PCa patients in a dual‐centre, two‐arm, open‐label, randomised Phase II trial (NCT04519879). This bidirectional translational research approach has supported the immunomodulatory role of WBM on MDSCs, thus strengthening the rationale for using WBM as a nutraceutical approach to slow the progression of prostate cancer.

We explored an alternative approach to target MDSCs in PCa by using the concept of ‘food as medicine’, specifically through WBM. It is important to note that WBM is not a ‘medicinal mushroom’, which is typically used in traditional cultural therapies, but rather a widely consumed food. Edible mushrooms, including *Agaricus bisporus* (WBM), *Grifola frondosa* (Maitake), and *Lentinus edodes* (Shiitake) mushrooms, have been reported to contain polysaccharides (β‐glucans) that have been shown to modulate the cellular immune response.^47^, ^48^ Preclinical investigations using animal models of cancer have reported that the injection of polysaccharide extracts of *Grifola frondosa* (Maitake)^49^, ^50^, ^51^ or *Lentinus edodes* (Shiitake) mushrooms^52^ may affect the immune system by reducing MDSCs. WBM has demonstrated immunoregulatory effects on DCs,^53^ NK cells,^54^ and other innate immune cells.^55^, ^56^ Our previously reported Phase I clinical trial in biochemical recurrence PCa patients showed that 4 responders among 36 participants who consumed WBM had a significant reduction in circulating MDSCs.^17^ The observed correlation between WBM consumption, reduction in MDSCs, and clinical responsiveness (PSA reduction) motivated us to design and undertake this full‐circle translational study. Our current research design included the use of murine models as well as immune cells from PCa patients under active surveillance who were part of our current Phase II clinical trial. Our ongoing Phase II trial (NCT04519879) incorporates a two‐arm, randomised design to include a control group, which will allow us to compare the efficacy and side effects of WBM more accurately. Our goal was to uncover the potential mechanisms by which oral WBM dietary intervention affects PCa progression and immune cell functions. We placed particular emphasis on MDSCs, utilising unbiased single‐cell RNA sequencing and multiplex flow cytometry for our analyses (Figure 8).

In preclinical studies, we used Lentinan (a purified β‐glucan from Shiitake mushrooms) as a reference botanical drug to the WBM extract based on the prior‐preclinical evidence that β‐glucan might be the major bioactive component in WBM inducing immune modulation.^55^, ^56^ According to the literature, β‐glucans account for 8–9% of WBM dry matter, 100 mg of WBM contains 8–9 mg of β‐glucans.^57^, ^58^ WBM extract may also contain proteins, lipids, and phytochemicals that could modify anti‐tumour immunity and/or exert direct cytotoxic effects on cancer cells,^59^ which should be further examined. In our Phase I trial involving PCa patients, we intentionally designed a dose‐escalation study with six different dosages (4, 6, 8, 10, 12, and 14 g/day). For chronic ingestion, 14 g/day was considered the highest practical dosage. The Phase I study did not aim to reach the maximum tolerated dosage (MTD) because our preclinical data as well as the Phase I clinical trial with PCa patients did not indicate a dose‐dependent effect.^17^ In the current study, a single dose of WBM tablets (14 g/patient/day) was provided to patients in the ongoing Phase II trial, while a single dose of WBM extract (6 mg/mouse/day) was administered to the animals. The dosage for the animals was based on the dosage provided to patients. For adult men with an average body weight of 70 kg, the daily intake of WBM powder in the Phase II trial is .2 g/kg/day (.2 mg/g/day). Considering that the average body weight of a mouse is 30 g, this corresponds to 6 mg/mouse/day. In addition, we used oral gavage as the route of administration to the mice because it closely mimics the natural consumption of WBM in humans, allowing for better absorption of putative active compounds and facilitating an accurate assessment of their systemic effects. Gavage also ensured a constant quantitative intake from time to time and animal to animal.^60^

Our animal model experiments confirmed that WBM consumption inhibits PCa progression (Figure 1). These effects were not direct on tumour cells but required an intact murine immune system (Figures 1 and S1). The major target cells of WBM appeared to be MDSCs at both the cellular and gene expression levels. WBM extract could minimise the suppressive activity of MDSCs against CD4^+^ T and CD8^+^ T cells, resulting in the restoration of the proliferative activity of both T cell types (Figure 2). As a result of the reduction in MDSC levels in mice, there was an increase in intratumoural T and NK cells in treated mice (Figures 3 and S4). These results were confirmed by nonbiased single cell analysis of PCa patient circulating immune cells and flow cytometry, which provide complementary evidence to our animal model studies. There was a decrease in MDSCs, accompanied by a concomitant increase in T and NK cell numbers and functions in the WBM treated patients, as there were no significant MDSC changes in the control patients (Figures 4 and S5). Single‐cell analyses of patients’ specimens, including clustering and annotation, revealed that a subset of MDSCs, notably PMN‐MDSCs, were particularly reduced in WBM‐treated patients (Figure 5). Functional enrichment analyses of these cells showed that gene clusters involved in the response to fungal infection (e.g., WBM) and regulation of the immune response were induced by WBM treatment (Figure 6). These studies in humans and animal models provide strong evidence for the role of MDSCs in response to WBM, a nutraceutical intervention. In addition to its possible use in enhancing the natural immune response in PCa, our experiments in animal models indicated that WBM can improve the response to immunotherapy. The combination of WBM with the immune checkpoint inhibitor PD‐1 enhances the effectiveness of the latter by extending tumour regression and prolonging animal survival time (Figure 7).

While our study has contributed valuable insights into the immunoregulatory activity of WBM in PCa, there are several limitations. First, we did not conduct a chemical analysis of the bioactive and bioavailable components present in the WBM extract. However, we believe that β‐glucan plays a significant role in the immunomodulatory activity of WBM. The challenge in the drug development of botanical products lies in their chemical heterogeneity, unlike traditional pipelines with well‐defined molecules.^61^ Acknowledging this complexity, the US FDA has offered regulatory guidance and a ‘totality‐of‐the‐evidence’ approach. This emphasises mechanism‐driven and clinically relevant testing, alongside standardisation in the development of botanical products, to ensure their reproducibility and therapeutic consistency.^62^, ^63^ Following the US FDA guidance, rather than focusing on characterising the chemical constituents of WBM, our approach prioritises biological and clinical responses to a standardised WBM preparation by testing its cytotoxicity and immune modulation activity before applying it to patients and animals.^60^ Second, there are differences between the animal disease models and human clinical trials. The syngeneic xenograft model in immunocompetent mice is widely acknowledged as a standard model for developing immune therapy and exploring immune‐oncological mechanisms.^64^ For our clinical target groups comprising patients with early‐stage low‐risk or biochemically recurrent PCa, an applicable murine model is absent.^65^ We aimed to mitigate these concerns by employing a two‐way translational study design and complementing the findings from murine models with data derived from human clinical trials. The murine models provide essential insights into biological mechanisms which could be validated through subsequent studies in patients treated with WBM. Third, the single‐cell analysis of whole blood from eight patients in our Phase II clinical trial confirmed changes in MDSCs, aligning with Phase I results and animal studies. These findings can be further validated by including data from more patients when their samples become available. These Phase II data will include clinical assessments of patients' tumour responsiveness assessed by radiological tests, histopathological evaluation, and gene expression profiling. Finally, our ongoing Phase II clinical trial is limited to PCa patients with early‐ and low‐risk PCa. Choosing the appropriate patient population was carefully considered in the trial design, requiring both scientific and ethical considerations. Natural molecules from botanical medications are typically not as potent as chemically synthetic drugs, which can lead to mild therapeutic effects. In terms of safety versus efficacy pertaining to patient selection, patients with low‐risk or low‐grade cancer that has a low risk of metastasising may benefit from using botanical products at the time of initial evaluation. The applicability of WBM for late stage, aggressive PCa or other cancer types remains unclear.^60^

In summary, our study involving both animal models and PCa patients elucidates the cellular and molecular mechanisms by which WBM consumption modulates the immune response in prostate cancer. It highlights the significant reduction of PMN‐MDSCs as a key biological response to WBM treatment, thereby enhancing anti‐cancer immunity through the activation of cytotoxic T and NK cells. Methodologically, our study established a representative model of a full‐circle translational study in developing botanical drugs by following the US FDA regulatory guidelines.^60^, ^61^, ^62^, ^63^ Further research and clinical investigations are needed to assess the optimal route of administration and dosage of WBM, along with other health impacts, such as effects on the human microbiota. Future studies should focus on uncovering the molecular mechanisms of the various mushroom constituents, their interactions with cancer immunotherapy, and their potential applications in clinical practice.

## 4 MATERIALS AND METHODS

### 4.1 Mushroom extract and other materials

White button mushroom (WBM) extract was prepared via hot water extraction, as previously described.^14^, ^15^, ^16^ Briefly, 6 g of freeze‐dried WBM powder, prepared from 60 g of fresh mushrooms purchased from a local market, was boiled in 1 L hot water for 3 h. The broth was centrifuged twice at 3000 × *g* for 30 min to collect the supernatant. The liquid fraction was rotor‐evaporated to dryness and then re‐dissolved in 1 mL of hot water to produce a 6× mushroom extract, containing 6 g of dried WBM powder/mL (6 g/mL). The extract was then used for both in vitro and in vivo mouse studies. Lentinan (purified β‐glucan from Shiitake mushrooms) was purchased from Carbosynth (Compton, Berkshire, UK) and used as a reference botanical drug. The cytotoxic and biological activities of WBM extract or Lentinan were evaluated with either PCa cells or immune cells. Antibodies and chemicals used in this study are listed in Tables S3 and S4.

### 4.2 Murine PCa cell lines and cytotoxicity assay

Murine PCa cell lines (TRAMP‐C2 and MyC‐CaP) were purchased from the American Type Culture Collection (ATCC) and were cultured in Dulbecco's modified Eagle's medium (DMEM) with 4 mM L‐glutamine, 1.5 g/L sodium bicarbonate, 4.5 g/L glucose, .005 mg/mL bovine insulin, 10 nM dehydroisoandrosterone, 5% Nu‐Serum IV, 5% fetal bovine serum (FBS), 1% penicillin and 100 µg/mL streptomycin. The cells were maintained in a mycoplasma‐free medium (LookOut® Mycoplasma PCR Detection Kit) at 37°C and 5% CO_2_.

The cytotoxicity of WBM extract and lentinan (a β‐glucan) on TRAMP‐C2 and MyC‐CaP was appraised using the MTS assay following a standard protocol. Briefly, cells were seeded in a 96‐well plate at a concentration of 1 × 10^5^ cells/mL for 24 h, followed by incubation with the WBM extract or lentinan for various durations (24–72 h). Concentrations of the WBM extract and lentinan ranged from 0 to 5 mg/mL for cancer cell treatments. Subsequently, MTS solution was added to each well, and the plates were incubated for 4 h. The absorbance was measured at 570 nm using a microplate reader.

#### 4.2.1 In vitro immune cells cytotoxicity and functional assay

Human monocyte‐like THP‐1 cells were used to determine the cytotoxicity and immune modulatory activity of WBM extract, compared to lentinan (a β‐glucan) as described.^21^, ^22^ Briefly, THP‐1 cells were seeded in a 24‐well plate at a concentration of 2 × 10^5^ cells/mL for 24 h and differentiated by phorbol 12‐myristate 13‐acetate (PMA, Sigma, St. Louis, MO, USA) for 3 days. Post differentiation, the culture medium was replaced with PMA‐free medium, and cells were grown for an additional 3 days. Differentiated THP‐1 cells were then stimulated with 10 µg/mL lipopolysaccharides (LPS; Sigma) as control. A total of 10–500 µg/mL WBM or β‐glucan was used for cytotoxicity assessment, and 0 to 50 µg/mL WBM or β‐glucan was used for immune modulatory activity screening. Treated cells were collected at specified time points and total RNA were extracted to measure the expression of TNF‐α, IL1B, IL6, and TLR2.

To investigate the effect of WBM on MDSCs, tumour‐infiltrating MDSCs (CD11b^+^Gr‐1^+^) were isolated from xenograft tumours in C57BL/6J mice using EasySep™ Mouse MDSCs isolation kits (STEMCELL, Seattle, WA, USA). Total RNA of isolated MDSCs was extracted to assess the expression of functional MDSC genes, including *Il1β, Il6, S100a8, S100a8, Stat3, Arg2*, *Nos2*, etc. To examine the effect of WBM on the suppressive function of MDSCs against T cell proliferation and activity,^3^ tumour‐infiltrating MDSCs (CD11b^+^Gr‐1^+^) were sorted by BD FACS Aria Fusion Cell Sorter. WBM was applied to co‐cultures of sorted MDSCs and with CD4^+^ and CD8^+^ T cells. Sorted tumour infiltrating MDSCs were co‐cultured with CFSE (a proliferation tracer) pre‐labelled CD4/CD8 cells at a ratio of 1:2. 10 µg/mL WBM extract was added to the co‐culture for 48 h. T cell proliferation was assessed by measuring the MFI of CFSE‐positive CD4/CD8 cells. In addition, given β‐glucans can induce differentiation and apoptosis of MDSCs,^20^ we also examined whether WBM extract, or β‐glucans (1 µg/mL to 10 µg/mL for 48 h) could induce MDSC apoptosis by using Annexin V‐APC/DAPI Apoptotic Assay Kit (ab236215, Abcam, Waltham, MA, USA) and/or reduce arginase 1 expression by quantitating cytoplasmic Arg1 fluorescence by flow cytometry.

#### 4.2.2 Mouse strains and PCa xenograft models

Immunocompromised (NSG) and immunocompetent mice (C57BL/6J and FVB) were obtained from Jackson Laboratories. They were housed in a pathogen‐free facility with a 12‐h light cycle in individually ventilated cages on corn cob bedding, with access to water and food ad libitum, at 22°C (range 21°C–24°C) and 50% humidity (range 35%–75%), with environmental enrichment and bedding material. Changes in body weight were monitored twice a week. Animal experiments were approved by the Institutional Animal Care and Use Committee (IACUC) of the City of Hope. TRAMP‐C2 (2.0 × 10^6^) or MyC‐CaP (1.0 × 10^6^) cells in PBS were injected into the flank of C57BL/6J or FVB mice, respectively, under isoflurane inhalational anaesthesia on a heat mat to aid recovery. The experimental group sizes were determined and approved by the regulatory authorities for animal welfare, with the defined tumour endpoint set at 600 mm^3^. In certain instances, this limit was exceeded on the last day of measurement, leading to immediate euthanasia of mice. The actual recorded tumour size, even when surpassing 1000 mm^3^, was documented and presented in both the article and source data files. All mouse experiments were conducted in strict accordance with protocols approved by the City of Hope Laboratory Animal Center.

#### 4.2.3 Prophylactic and therapeutic treatments with WBM extracts in murine model

To evaluate the antitumour effects of WBM, we used both prophylactic and therapeutic treatment models. In the prophylactic treatment model, C57BL/6J or FVB mice were subjected to pretreatment with WBM extract (6 mg/day), β‐glucan (1 mg/day) in PBS, or PBS alone (Control/Ctrl) as the vehicle control via oral gavage daily, followed by tumour cell injection of TRAMP‐C2 or MyC‐CaP tumour cells after 7 days pre‐treatment. Day 0 of tumour measurement was the day of cell injection. The palpable tumour size was monitored twice per week and calculated using the following equation: (length × width^2^)/2. In the therapeutic treatment model, C57BL/6J mice were injected with TRAMP‐C2 tumour cells. When the tumours reached 250 mm^3^, the mice were assigned to the treatment or control group and received daily oral gavage of WBM or PBS, respectively. Day 0 of tumour measurement was the day of treatment initiation. Tumour size and body weight were recorded twice per week until the treatment endpoint. For experiments to generate tumour growth and survival curves, the endpoint for determining survival rate was set at a tumour size of 600 mm^3^, and a size of 1000 mm^3^ was used as the criterion for euthanasia. For experiments to collect tumours/spleen/blood/tumour‐draining lymph nodes (TDLNs) for immune cell phenotype, differential gene expression, and histological analysis, the mice were euthanised on the same day when the tumour size between treatments and control groups was statistically different. The tumours/spleen/TDLNs were mechanically processed into single‐cell suspensions, and cell viability and counts were evaluated. Single‐cell extracts from these tissues were frozen immediately in 20% DMSO with 80% FBS and stored in ‐80°C for immune‐phenotyping assay. Mice were excluded from the analyses if pre‐established exclusion criteria were met, such as tumour ulceration as defined by the animal protocol endpoint, or instances where no tumour growth was observed.

To determine the potential cytotoxic effect and dependence of the immune‐mediated antitumour effect of WBM extract/β‐glucan on xenograft tumours, we also implanted TRAMP‐C2 cells in the right flank of immunodeficient NSG mice. NSG mice received WBM (6 mg/day), β‐glucan (1 mg/day), or PBS daily for 7 days before tumour cell inoculation. Tumour size and body weights were measured twice a week until the tumours reached the maximum volume (1000 mm^3^) as the endpoint of the experiments.

#### 4.2.4 Intra‐tumoural immune cell phenotyping in WBM‐treated xenograft tumours

For the multiplex flow cytometry assay, xenograft tumours were mechanically dissociated into 2–3 mm pieces and incubated with a 5 mL digestion cocktail consisting of 400 µL of 5 g/mL collagenase IV (Worthington, LS004189) and 5 U/mL DNase I (195 U/mL) (Invitrogen, 1928344) in Hanks Balanced Salt Solution. The resulting mixture was passed through a 70 µm nylon cell strainer. Cell suspensions were generated using the gentleMACS™ Dissociator (Miltenyi Biotec, San Diego, CA, USA). Red blood cells were removed using lysis buffer (Invitrogen, 00‐4333‐57). Subsequently, the cell suspensions were incubated for 5 min at room temperature with purified anti‐mouse CD16/CD32 Fc block (BioLegend, 156604) before the addition of cell surface antibodies. An eBioscience Intracellular Staining Kit (eBioscience, 88‐8824‐00) was used; the antibodies used for immunostaining are listed in Table S4. Data acquisition was performed using BD Accuri C6 Plus, and analysis was conducted using FlowJo v10.0. An increased proportion of cells within each tumour expressing a specific combination of immune cell‐specific cell surface markers was considered indicative of an increased proportion of immune cells.

For multiplex IHC experiments, xenograft tumours were collected and fixed with paraformaldehyde. Multiplex immunohistochemistry (IHC) analysis was performed by the Research Pathology Core of the City of Hope. The specific isotype control (Rabbit IgG, monoclonal (EPR25A)‐Isotope control, ab172730) was used to ensure specific antibody binding. The antibodies used for immunostaining are listed in Table S4. Images were captured using an Olympus BX46 microscope equipped with a DP27 camera. Representative images were taken at 10× and 40× objectives, with scale bars of 400 µm and 100 µm, respectively. The 10× images were utilised to quantify the counts of tumour infiltrating CD4, CD8 T cells, and MDSCs, and to illustrate the spatial distribution pattern of those cells. CD4‐positive, CD8‐positive, and Gr‐1‐positive cells were scored via the Cell Detection and Cell Classification functions implemented in QuPath software. Briefly, digital images of the stained slides were uploaded onto QuPath, where annotations for immune cells were made. The annotations created targeted immune cells classifiers, which were applied to the region of interest (ROI). Detected cells within the ROI were classified as positive cells versus negative cells. Calculations and reports were made for the total number of cells, the number of positive cells, and the proportion of positive cells relative to the total cell count.

#### 4.2.5 Intra‐tumoural immune gene expression profiling in WBM‐treated xenograft tumours

Immune gene expression in xenograft tumours was assessed using NanoString® PanCancer Immuno‐Oncology 360TM on the nCounter® SPRINT platform (NanoString® Technologies, Seattle, WA, USA). This specialised 770‐plex murine gene expression panel, designed to profile the immune system and tumour microenvironment, was employed to characterise individual genes and pathways influencing tumour‐immune interactions. Three xenograft tumours from the control and WBM‐treated groups were subjected to gene expression assay. The expression levels of 770 immune‐related genes and immune function/pathway scores were compared between PBS and WBM groups. The nSolver 4.0 software (NanoString Technologies) was employed to calculate pathway scores and cell abundance scores based on a previously reported methodology.^24^ Immune function/pathway scores were calculated as the average log2 normalised expression of selected marker genes. The immune cell types were determined using the microenvironment cell population counter (MCP‐counter) method based on the expression levels of cell type‐specific marker genes.^24^ The immune function/pathway score and the immune cell type score obtained from nSolver Advanced Analysis were analyzed using GraphPad Prism software. *t*‐Tests with Welch's correction were applied for comparisons between groups, with significance defined as *p* < .05. NanoString analysis was conducted by the Molecular Pathology Core at the City of Hope, and the resulting data file in RCC format was used for further analysis.

#### 4.2.6 Peripheral immune cell phenotyping in WBM‐treated mice

Leukocytes in whole blood were obtained following lysis of red blood cells. Spleen and TDLNs were dissociated into cell suspensions using a gentleMACS™ Dissociator (Miltenyi Biotec, San Diego, CA, USA) in RPMI1640 medium. Red blood cells were subsequently removed using a lysis buffer (Quality Biological, 118‐156‐101). Leukocytes from the blood, spleen, and TDLNs were characterised using multiplex flow cytometry to identify various immune cell types including MDSCs, T cells, NK cells, and DCs. A detailed list of antibodies used for immune cell staining is shown in Table S3.

#### 4.2.7 Peripheral immune cell phenotyping in whole blood specimens from PCa patients treated with WBM

Peripheral immune cell phenotyping using multiplex flow cytometry was conducted in 18 patients (10 WBM tablet‐treated patients and 8 untreated Control patients) who participated in the randomised Phase II clinical trial (NCT04519879) and were diagnosed with low‐risk PCa under active surveillance. The inclusion and exclusion criteria for patient recruitment were designed to minimise the impact of confounding factors such as diet, lifestyle, and concurrent medications on the WBM intervention. Participants were instructed to maintain consistent diets and lifestyles throughout the trial period, and detailed records of concurrent medications were collected. Additionally, we conducted regular assessments to monitor compliance for WBM intake. All patients provided written informed consent and the study was approved by the IRB Committee of the City of Hope.

Frozen‐preserved whole blood samples collected at baseline and after 3 months of treatment from these 18 patients were processed for immune cell phenotyping using multiplex flow cytometry following a previously reported protocol.^66^ Intact red blood cells were lysed (Quality Biological, 118‐156‐101). The cell suspensions were incubated for 5 min at room temperature with a purified anti‐mouse CD16/CD32 Fc block (BioLegend, 156604) before the addition of cell surface antibodies. An eBioscience Intracellular Staining Kit (eBioscience, 1999385) was used; the antibodies used for immunostaining are listed in Table S4. Data acquisition was performed using a BD LSR Fortessa X‐20 Cell Analyzer and the analysis was conducted using FlowJo v10.0.

#### 4.2.8 Single immune cell profiling of blood specimens from PCa patients under WBM treatment

Eight patients diagnosed with low‐risk prostate cancer (PCa) under active surveillance were selected for single‐cell profiling analysis. Written informed consent was obtained from all the patients, and the study was approved by the Ethics Committee. Single immune cell profiling was performed using Integrative Genomics Core of the City of Hope. Paired whole blood samples from eight WBM‐treated patients at baseline and after 3 months of treatment were collected, at two time periods, and processed based on a single immune cell profiling protocol. Leukocytes from whole blood were enriched according to the 10X Genomics standard protocol.^67^ The cell viability of all tested samples exceeded 80%. As shown in Table S3, leukocytes of former four patients (Discovery Cohort, patients 1 to 4) were converted to barcoded scRNA‐seq libraries using the Chromium Next GEM Single Cell 5′ v2 Reagent (10X Genomics, 120237) to prepare gene expression library and TCR/BCR repertoire library. Leukocytes of the latter four patients (Validation Cohort, patients 5 to 8) were processed with Chromium Next GEM Single Cell 3ʹ v3.1(10X Genomics, 1000121) to prepare gene expression library only. The libraries were then sequenced using NovaSeq 6000 at an average depth of approximately 50 000 reads per cell. The FastQC software was used for quality checks. Cell Ranger software (version 37.1.0) was used for the initial processing of sequencing data to derive gene expression profiles. Neutrophils, which express only a few hundred genes, were enriched according to the 10X Genomics Guild Line with intron mode enabled when running CellRanger.^68^

After obtaining the gene counts for individual samples, the counts were aggregated to create a gene‐barcode matrix for four paired Baseline versus 3‐month treatment samples. This matrix was then used to create a Seurat object using Seurat package v4.0 (https://satijalab.org/). The cells were filtered based on the following criteria: gene number between 200 and 8000, UMI count > 500, and mitochondrial content less than 15%. Following filtration noted in Table S3, for Discovery dataset, a total of 18 982 cells (3077/1925/8441/5539) from baseline samples and 12 668 cells (3754/2652/3617/2645) after 3‐month treatment samples, using the Chromium Next GEM Single Cell 5′ v2 Reagent (10X Genomics, 120237), were retained for subsequent analysis. For Validation dataset, a total of 35 378 cells (8805/4642/9541/12 390) from baseline and 37 883 cells (11 875/7281/8415/10 312) from 3‐month treatment samples, the Chromium Next GEM Single Cell 3ʹ v3.1 (10X Genomics, 1000121), were submitted for downstream analysis. Since two datasets were prepared with different library prep kits at different times, we employed a discovery‐validation design. The former 4 patients (8 samples) were used as the Discovery dataset and the latter 4 patients (8 samples) as the Validation dataset. This approach allowed us to conduct an unbiased discovery‐validation analysis with minimal batch effects from two independent datasets.

Cells were clustered using the Seurat package at a resolution of .5. Cell clustering and annotation were performed using an integrated approach involving automated and manual methods along with a custom gene set enrichment strategy. Initial cell‐type predictions were generated from Azimuth by mapping the query datasets to annotated human peripheral blood cell references in Seurat.^69^ Manual annotation was applied to identify neutrophils using a previously described cell and tissue marker gene database.^70^ MDSCs were manually annotated by referring to a previously described MDSC‐specific gene signature marker,^33^ and MDSC signature scores were calculated for monocyte and neutrophil subclusters to assess the resemblance of MDSC clusters within these two major cell types.^33^ Compositional analysis was conducted at the level of cell identity clusters corresponding to known cell types or states related to the WBM treatment. Differential expression analyses were performed on average (pseudobulk) gene expression for the final 35 annotated cell populations across samples. Differentially expressed genes were defined based on an empirical Bayes moderated *t*‐test with *p* < .05 (false‐discovery rate corrected) and > 1.5‐fold change between baseline and 3‐month of treatment. Pearson's correlation was used to identify genes with log2 expression correlated with donor age (log2 normalised) with a correlation coefficient exceeding .6 or falling below –.6. Functional enrichment analyses were performed using clusterProfiler v4.0.^71^

### 4.3 Combination treatments of WBM extracts and anti‐PD‐1 in mice

In the experiment involving the combination treatment of WBM extract with immune checkpoint inhibitors (anti‐PD‐1), wild‐type C57BL/6J mice were categorised into four groups of 10 males each: PBS/Isotype, PBS/anti‐PD‐1, WBM/Isotype, and WBM/anti‐PD‐1. The dosage and frequency of WBM extract administration were the same as those previously described. Anti‐PD‐1 was administered intravenously twice per week at a dose of 50 µg per mouse.^72^ The tumour size and body weight of the mice were monitored every 2 days.

#### 4.3.1 qRT‐PCR

RNA isolation was conducted using the RNeasy Plus Mini Kit (QIAGEN, 74134) following the manufacturer's instructions. Complementary DNA (cDNA) was synthesised using either an iScript cDNA Synthesis Kit (Bio‐Rad, 1708841) or a High‐Capacity RNA‐to‐cDNA Kit (Applied Biosystems, 4387406). Quantitative PCR (qPCR) was performed using the PerfeCTa SYBR Green FastMix (Quantabio, 95072–250) and gene‐specific primers for the genes of interest (Table S6) in a CFX384 Real‐time PCR detection system (BioRad). To compare target mRNA expression under different conditions, we correlated the copy numbers of each gene with the total RNA concentration, expressing qPCR values as mRNA copies/µg of total RNA. For comparing gene expression within the same cell/tissue type, the target mRNA expression was quantified using the ΔΔCt method, normalising to Gapdh transcript levels.

#### 4.3.2 Statistical analysis

The results are expressed as mean ± SEM. Statistical analysis involved a two‐tailed Student's *t*‐test or Mann–Whitney *U* test, as deemed appropriate. Multiple group comparisons were performed using one‐way or two‐way ANOVA, followed by Tukey's or Sidak's multiple comparison tests. Statistical analyses were conducted using the GraphPad Prism software (GraphPad Inc., La Jolla, CA, USA), and significance was defined as *p* < .05.

## AUTHOR CONTRIBUTIONS

X.W., S.M., and S.C. designed this study. X.W. and S.M. both contributed equally to this work. X.W., Y.C., and K.W. performed experiments. X.W., S.X., and S.M. performed flow cytometry sorting and analyses. X. Wu and J.W. performed the sequencing. X.W. and X. Wu performed bioinformatic analyses. P.T. and C.L. recruited clinical trial participants. S.C. supervised the study. X.W., S.M., Y.C., K.W., and S.C. drafted and revised the manuscript. All the authors have read and approved the final manuscript.

## CONFLICT OF INTEREST STATEMENT

The authors declare that there is no conflict of interest.

## ETHICS STATEMENT

The animal experiments performed in this study were approved by the Institutional Animal Care and Use Committee of the City of Hope and were performed according to the institutional and National Institutes of Health guidelines for animal care and use.

The human specimens collected in this study were approved by the Institutional Review Board (IRB) of the City of Hope, and informed consent was obtained from each human subject before specimen collection.

## Supporting information



Supporting Information

## Data Availability

The scRNA‐seq data generated in this study are publicly available in the Gene Expression Omnibus (GEO) at GSE266985 for the Discovery dataset and at GSE275574 for the Validation dataset.
