# Supplementary material for: Reduction of myeloid‐derived suppressor cells in prostate cancer murine models and patients following white button mushroom treatment
Source: Clin Transl Med. 2024 Oct 10;14(10):e70048. doi: 10.1002/ctm2.70048 (PMC11467013; doi:10.1002/ctm2.70048)
Supplement: Supplementary file 1 — Supporting Information [file CTM2-14-e70048-s001.docx]

**Fig. S1: WBM Extract Slows Tumor Growth in TRAMP-C2/MyC-CaP Flank Tumor Xenografts in C57BL/6J and FVB Mice Without Evident Toxicity. A.** The tumor growth curve for subcutaneous MyC-CaP flank tumor xenografts in FVB mice, treated with PBS, WBM extract, or β-glucan is shown (PBS: n=4; WBM: n=5, 6 mg/mouse/day; β-glucan: n=5, 1 mg/mouse/day; repeated 3 times). **B.** The bar graph shows the average tumor weight measurements from the treatment groups, which is accompanied by an image of the subcutaneous MyC-CaP flank tumor xenografts from the FVB mice. The line graphs show body weight monitoring of **C.** C57BL/6J mice and **D.** FVB mice treated with PBS, WBM extract, or β-glucan. The bar graphs depict cell viability assessments for **E.** TRAMP-C2 cells and **F.** MyC-CaP cells treated with PBS, WBM extract (1 ~ 5 µl/ml, repeated 3 times), or β-glucan (1 ~ 5 mg/ml, repeat 3 times). Data are presented as mean tumor volume, body weight, or cell viability ± SEM and were analyzed using ordinary one-way ANOVA with Tukey’s post-test for multiple comparisons. Significance notations: ns (non-significant), ** p<0.01, *** p<0.001.


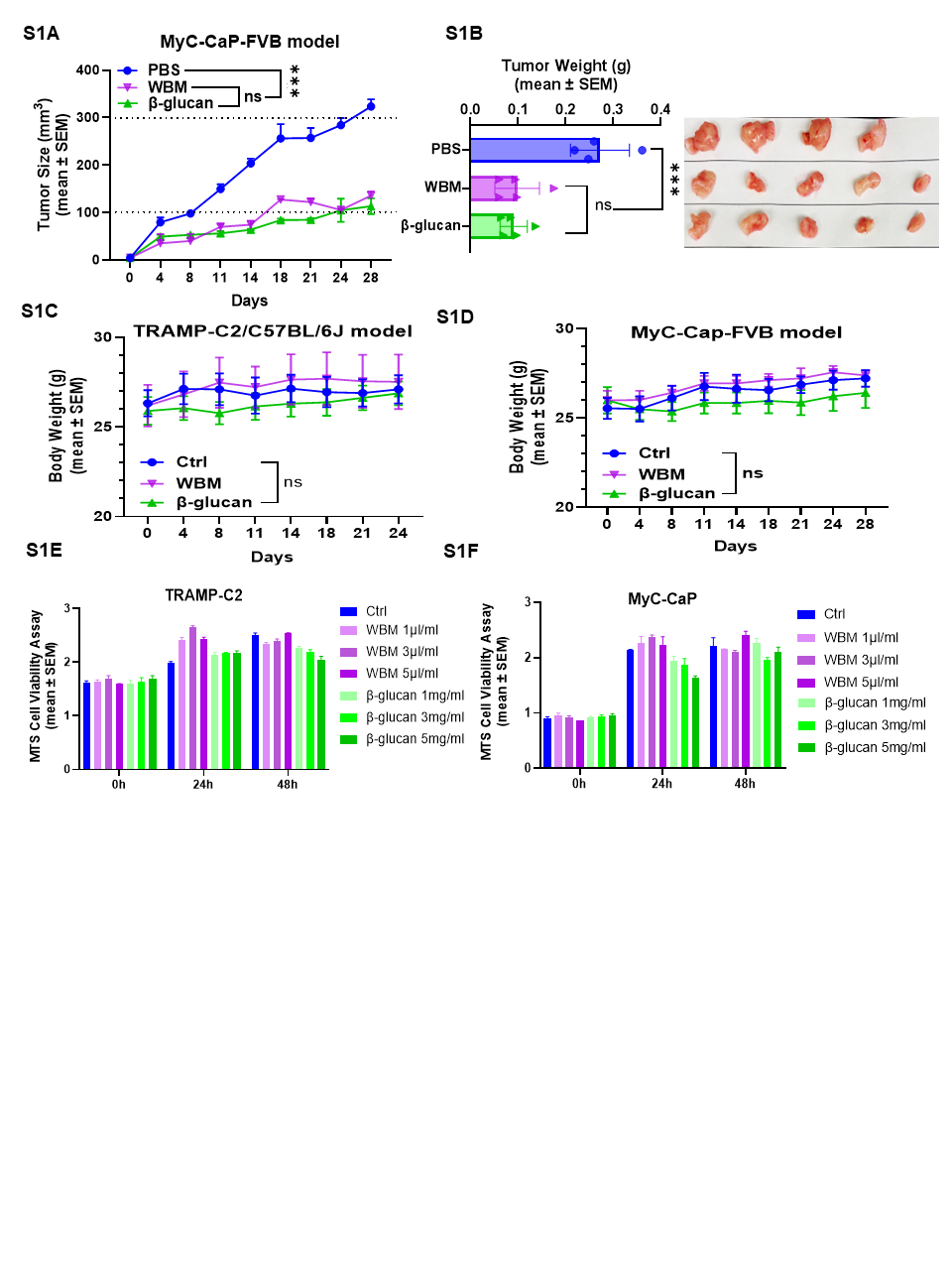


**Fig. S2: Reduction of Tumor-Infiltrating MDSCs in TRAMP-C2 Flank Tumor Xenografts in C56BL/6J Mice Following WBM Extract Treatment. A.**  The representative IHC staining images (scale bar = 100 μm, n=4) showing Gr-1 positivity in tumors. **B.** Human monocyte-like THP-1 cells were used to evaluate the cytotoxicity induced by WBM extract or lentinan (β-glucan used as reference) (10 ~ 500 µg/ml, repeated 3 times) and **C.** immune modulatory activity of WBM extract or lentinan (β-glucan used as reference) (5 ~ 50 µg/ml, repeated 3 times). **D.** Apoptotic MDSCs following treatment with PBS, WBM extract, or β-glucan (1 and 10 µg/ml, repeated 3 times) were detected and analyzed using flow cytometry. **E.** Analysis of Arginase1 (Arg1) positivity in MDSCs post-treatment with PBS, WBM extract, and β-glucan (1 and 10 µg/ml, repeated 3 times) was achieved through flow cytometry. Significance notations: ns (non-significant), ** p<0.01, *** p<0.001.


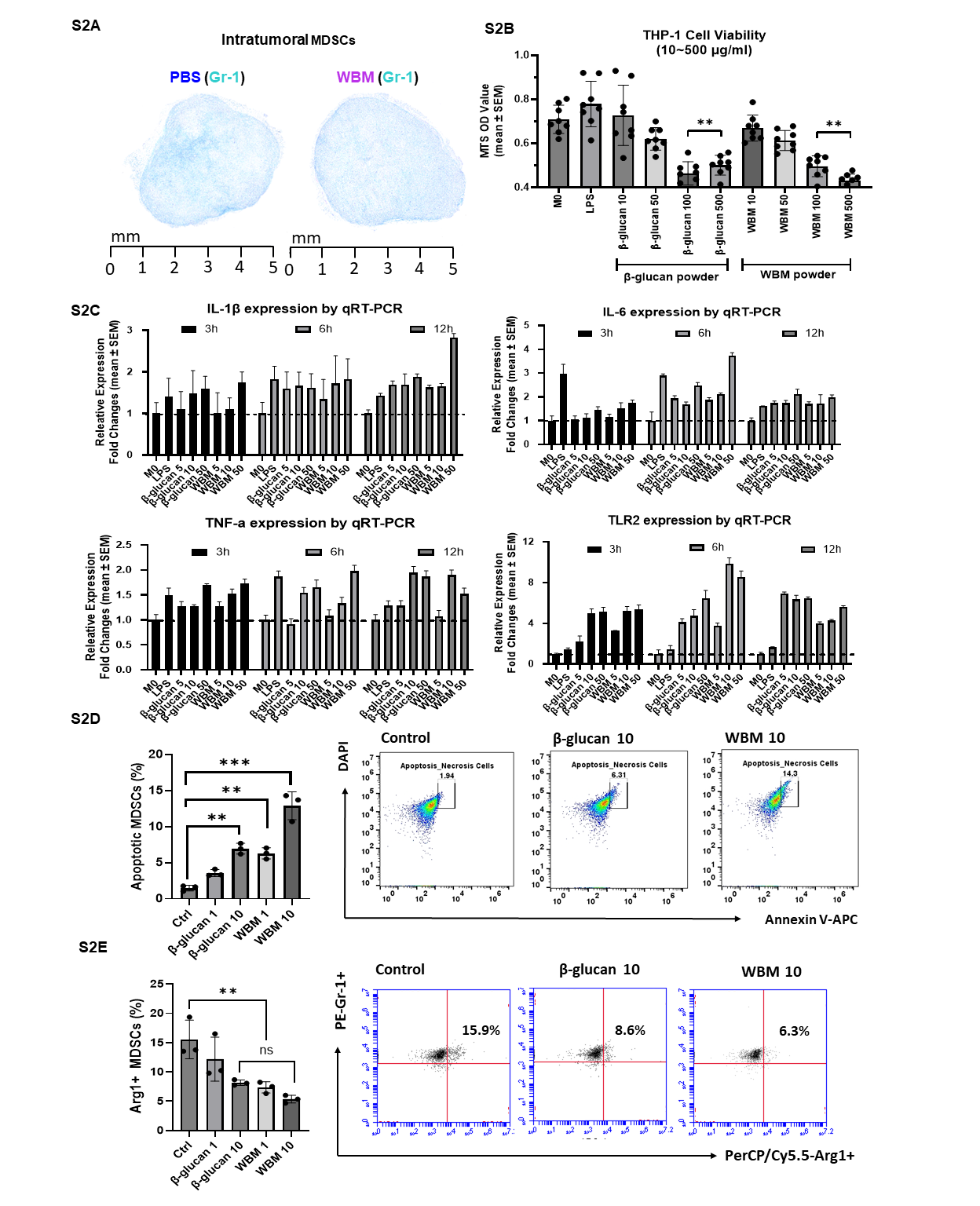


**Fig. S3: WBM Extract Modifies T Cell Populations within TRAMP-C2-C57BL/6J Mice Model. A**. Multiplex IHC staining images (scale bar = 100 μm, n= 4) show CD4^+^ T cells (teal) and CD8^+^ T cells (yellow) within tumors. **B.** The pairwise box plots show white blood cell counts from blood samples of mice treated with PBS (n = 5) or with WBM extract (n = 5), which were measured using the Sysmex XN-1000 hematology analyzer. **C.** Circulating CD4^+^ and CD8^+^ T cells, **D.** CD4^+^ and CD8^+^ T cells from spleens, and **E.** CD4^+^ and CD8^+^ T cells from tumor-draining lymph nodes (TDLNs) were identified through flow cytometry analysis. The bar graphs are quantitative comparisons of blood samples from mice (n = 5) treated with PBS or WBM. The data are represented as mean ± SEM and were analyzed using ordinary one-way ANOVA with Tukey’s post-test for multiple comparisons. Significance notations: ns (non-significant), *p<0.5, **p<0.01, ***p<0.001, ****p<0.0001.


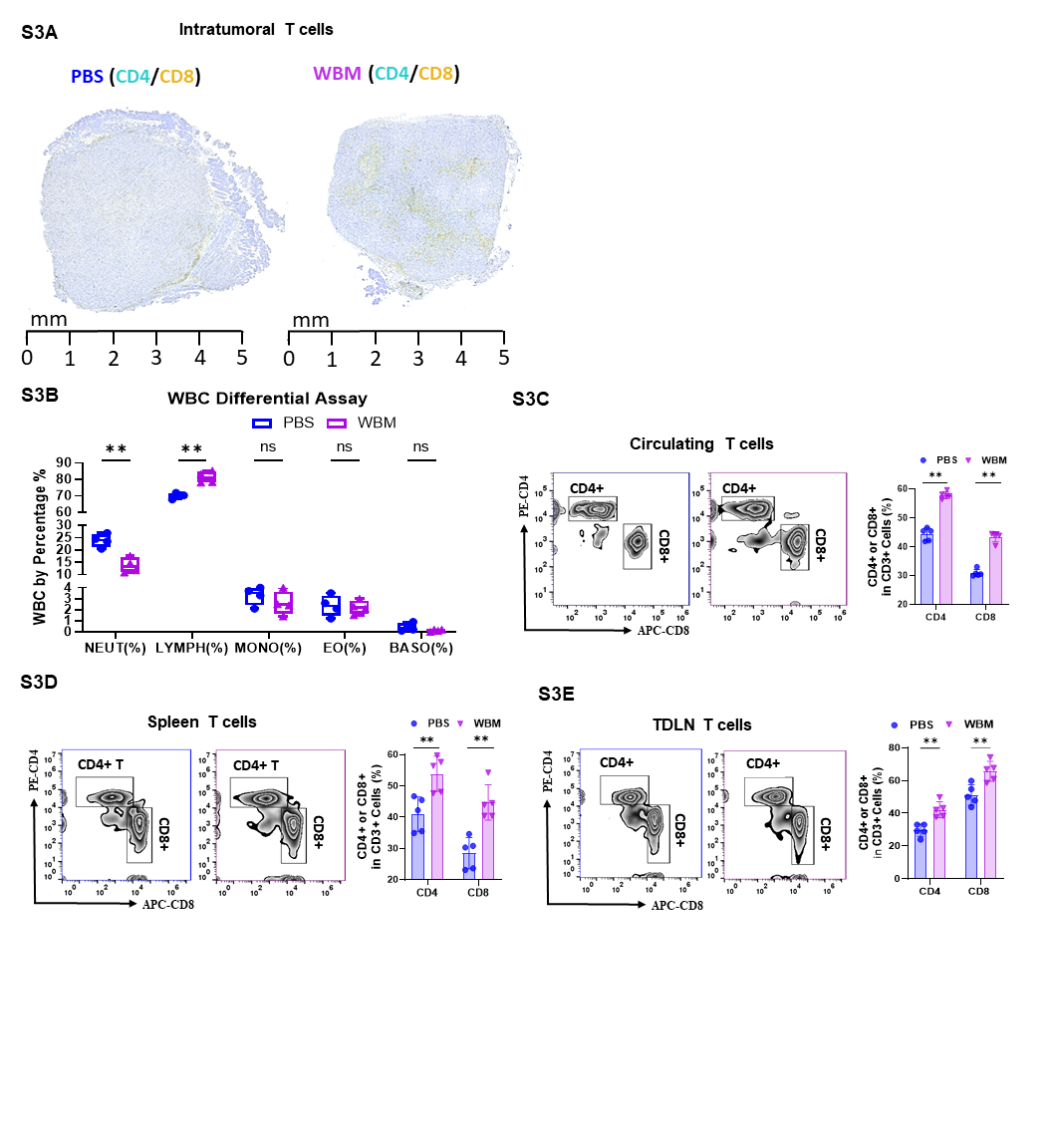


**Fig. S4: Changes in Intratumoral Leukocytes in TRAMP-C2 Flank Tumor Xenografts in C57BL/6J Mice Following WBM Extract Treatment. A**. The bar graph summarizes immune cell scores in tumors of PBS-treated (n=3) vs. WBM-treated (n=3) groups, which was determined using the NanoString nSolver^TM^ analysis program. The representative flow cytometry results for **B.** intratumoral dendritic cells (DCs), **C.** intratumoral macrophages, **D.** intratumoral T-regulatory (Treg) cells, and **E.** intratumoral natural killer (NK) cells are accompanied by bar graphs to summarize quantitative analyses comparing blood from mice from the PBS/WBM (n=5) treatment groups. The data are presented as mean value ± SEM and were analyzed using ordinary one-way ANOVA with Tukey’s post-test for multiple comparisons. Significance notations: ns (non-significant), *p<0.5, **p<0.01, ***p<0.001, ****p<0.0001.


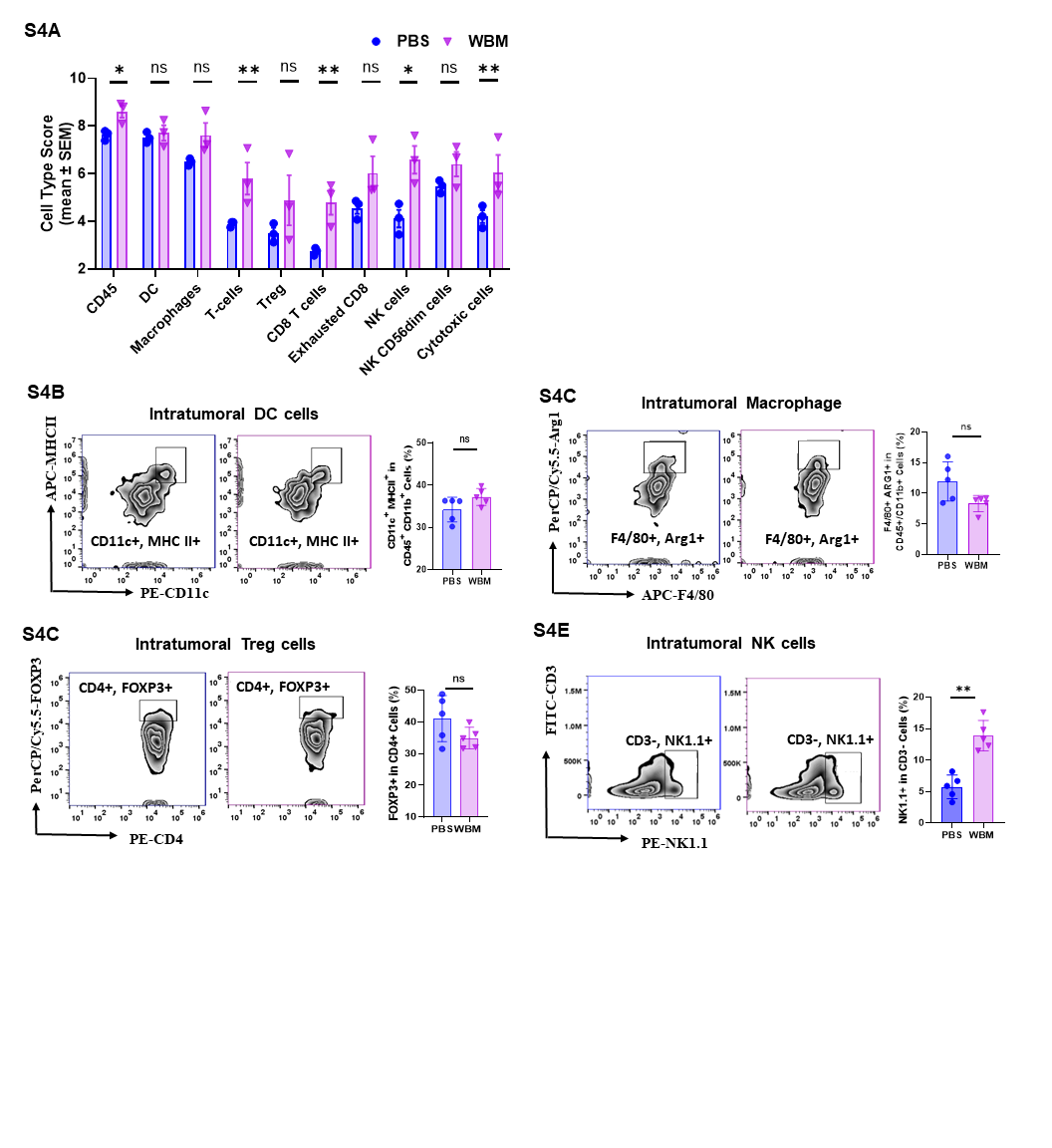


**Fig. S5: Changes in Circulating MDSCs in PCa Patients without WBM Consumption.** The representative flow cytometry results identify circulating M-MDSCs (CD11b^+^/CD14^+^) and PMN-MDSCs (CD11b^+^/CD15^+^). The pairwise line-point scatter plot summarizes the quantitative cell number analysis which examined the patients' (n=8) blood samples at baseline (first day of enrollment) and at the 3-month follow-up period without WBM treatment. The data is presented utilizing pairwise line-spot plots and were analyzed using ordinary one-way ANOVA with Tukey’s post-test for multiple comparisons. Significance notations: ns (non-significant), *p<0.5, **p<0.01, ***p<0.001, ****p<0.0001.


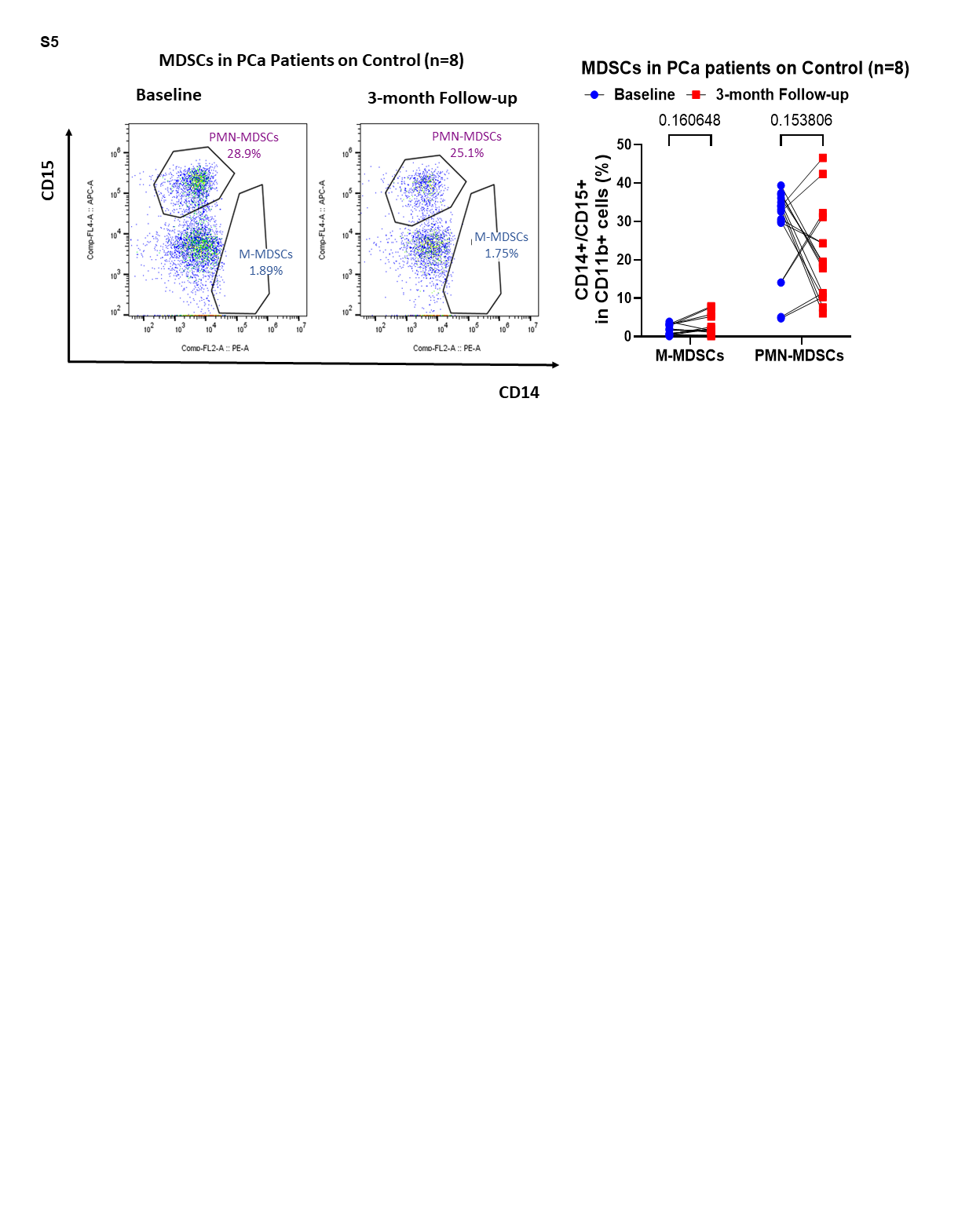


**Fig. S6: Activation of Circulating T Cells in PCa Patients with WBM Consumption. A.** The pairwise line-point scatter plot shows the quantitative analysis of CD4^+^ (TNFα/IFN-γ positive) and CD8^+^ (TNFα/IFN-γ/Granzyme B) cells in the patients' (n = 10) blood samples at pre-treatment (day of enrollment) and post-treatment (3-month WBM treatment). The representative flow cytometry results show differences in levels of **B.** circulating CD4^+^ T cells (CD3^+^/CD4^+^) and CD8^+^ T cells (CD3^+^/CD8^+^), **C.** TNFα/IFN-γ double-positive CD4^+^ cells, **D.** TNFα/IFN-γ double-positive CD8^+^ cells and **E.** Granzyme B positive CD8^+^ cells in samples of pre- and post-treatment. The data are shown using pairwise line-spot plots and were analyzed using ordinary one-way ANOVA with Tukey's post-test for multiple comparisons. Significance notations: ns (non-significant), *p<0.5, **p<0.01, ***p<0.001, ****p<0.0001.


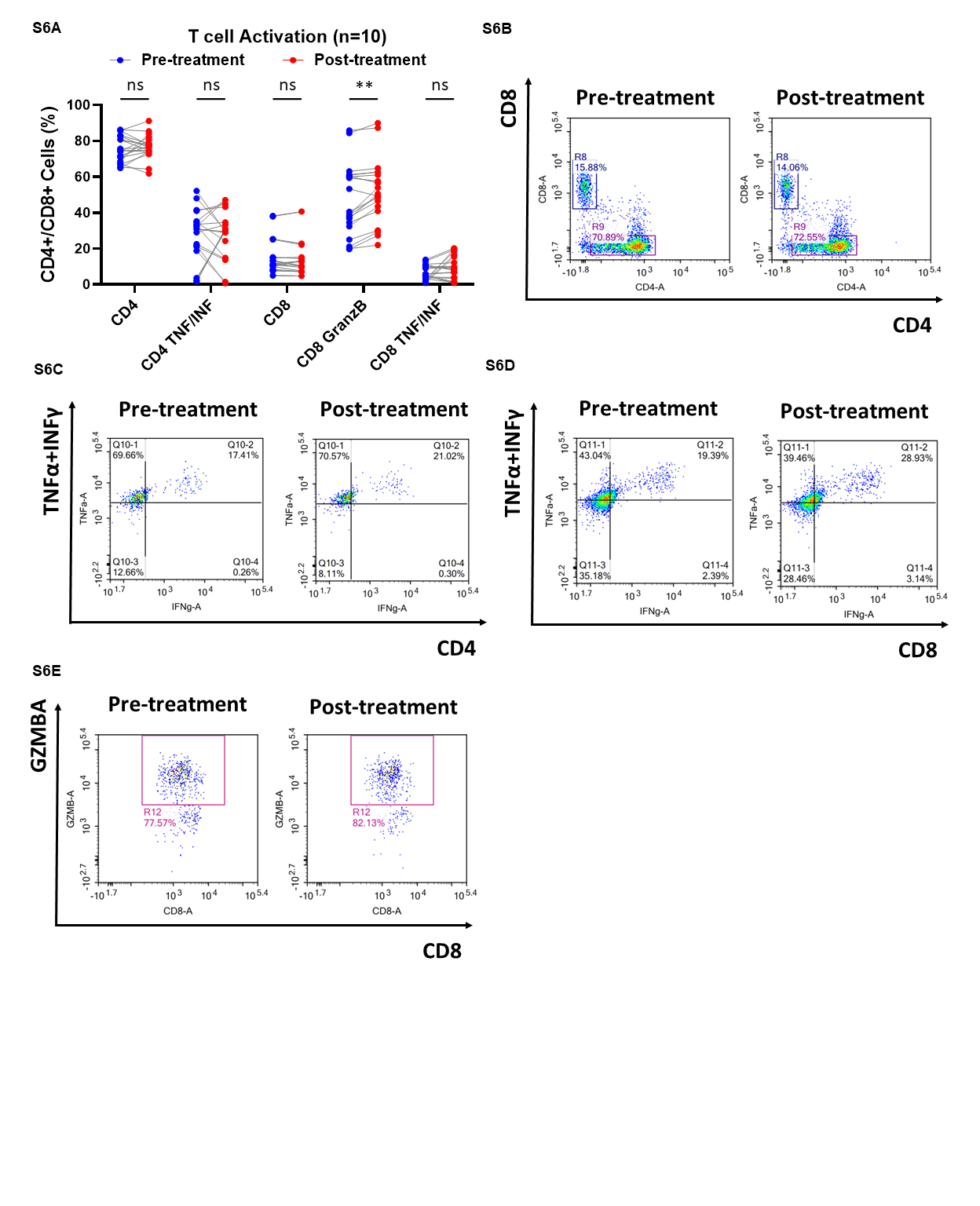


**Fig. S7: Immune-Checkpoint Expression in Circulating T Cells in PCa Patients with WBM Consumption. A.** The pairwise line-point scatter plot shows the quantitative analysis of CD4/CD8 (PD-1, CTLA-4, and TIM3) cells in patients' (n = 10) blood samples pre-treatment (day of enrollment) and post-treatment (3-month WBM treatment). The representative flow cytometry results show pre- and post-treatment levels of **B.** PD-1 and TIM3 double-positive CD4^+^ T cells, **C.** PD-1 and CTLA-4 double-positive CD4^+^ T cells, **D.** PD-1 and TIM3 double-positive CD8^+^ T cells, and **E.** PD-1 and CTLA-4 double-positive CD8^+^ T cells. Data are presented as a pairwise line-spot plot and were analyzed using ordinary one-way ANOVA with Tukey's post-test for multiple comparisons. Significance notations: ns (non-significant), *p<0.5, **p<0.01, ***p<0.001, ****p<0.0001.


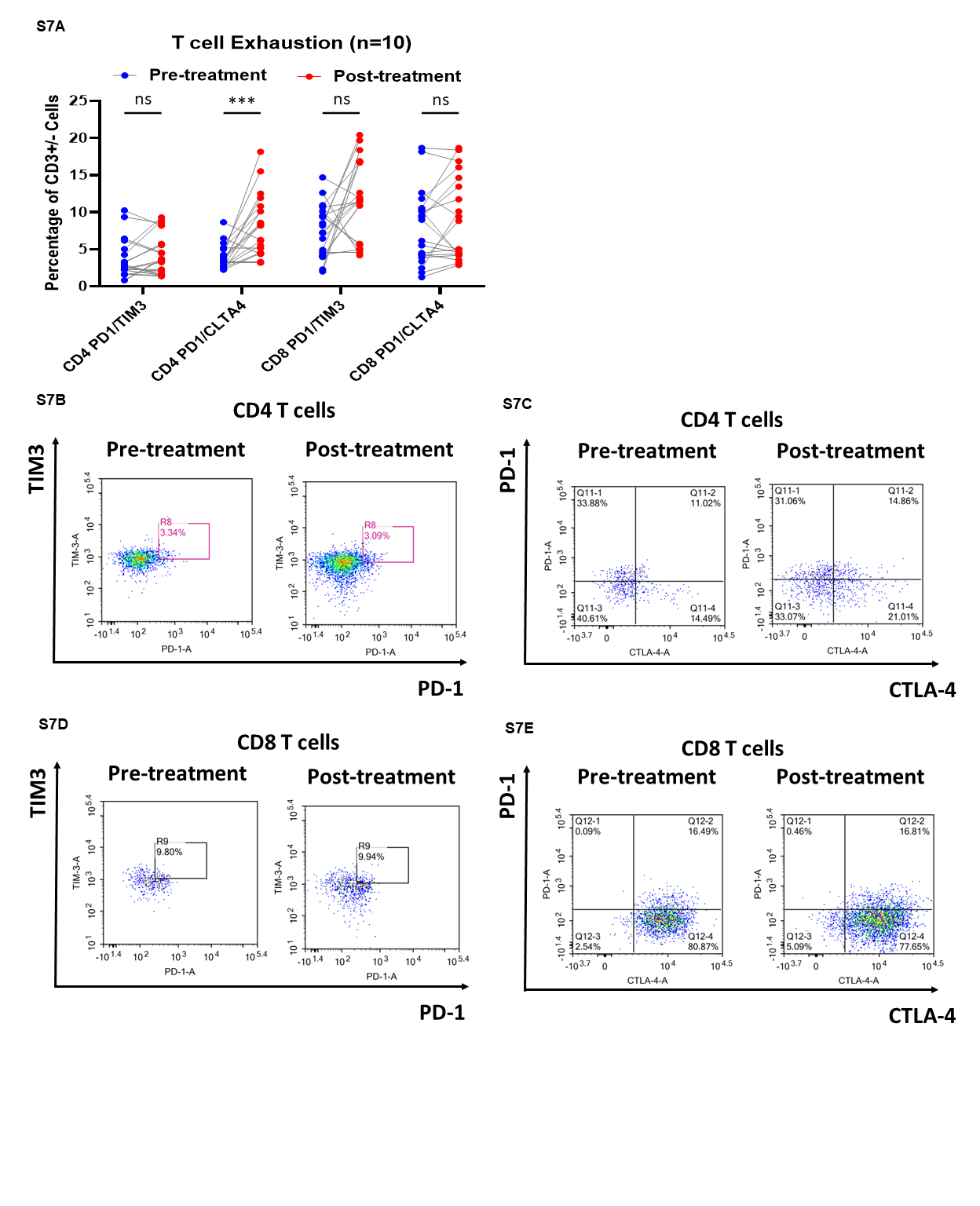


**Fig. S8: Activation and Immune-Checkpoint Expression in Circulating NK Cells in PCa Patients with WBM Consumption. A.** The pairwise line-point scatter plot quantifies the amount of NK cells positive for TNFα, IFN-γ, Granzyme B, TIGIT, and NKG2A in patients' (n = 10) blood samples pre-treatment (day of enrollment) and post-treatment (3-month WBM treatment). The representative flow cytometry results show **B.** TNFα/IFN-γ positive NK cells and **C.** TIGIT & NKG2A double-positive NK cells. The data are shown using pairwise line-spot plots and were analyzed using ordinary one-way ANOVA with Tukey's post-test for multiple comparisons. Significance notations: ns (non-significant), *p<0.5, **p<0.01, ***p<0.001, ****p<0.0001.


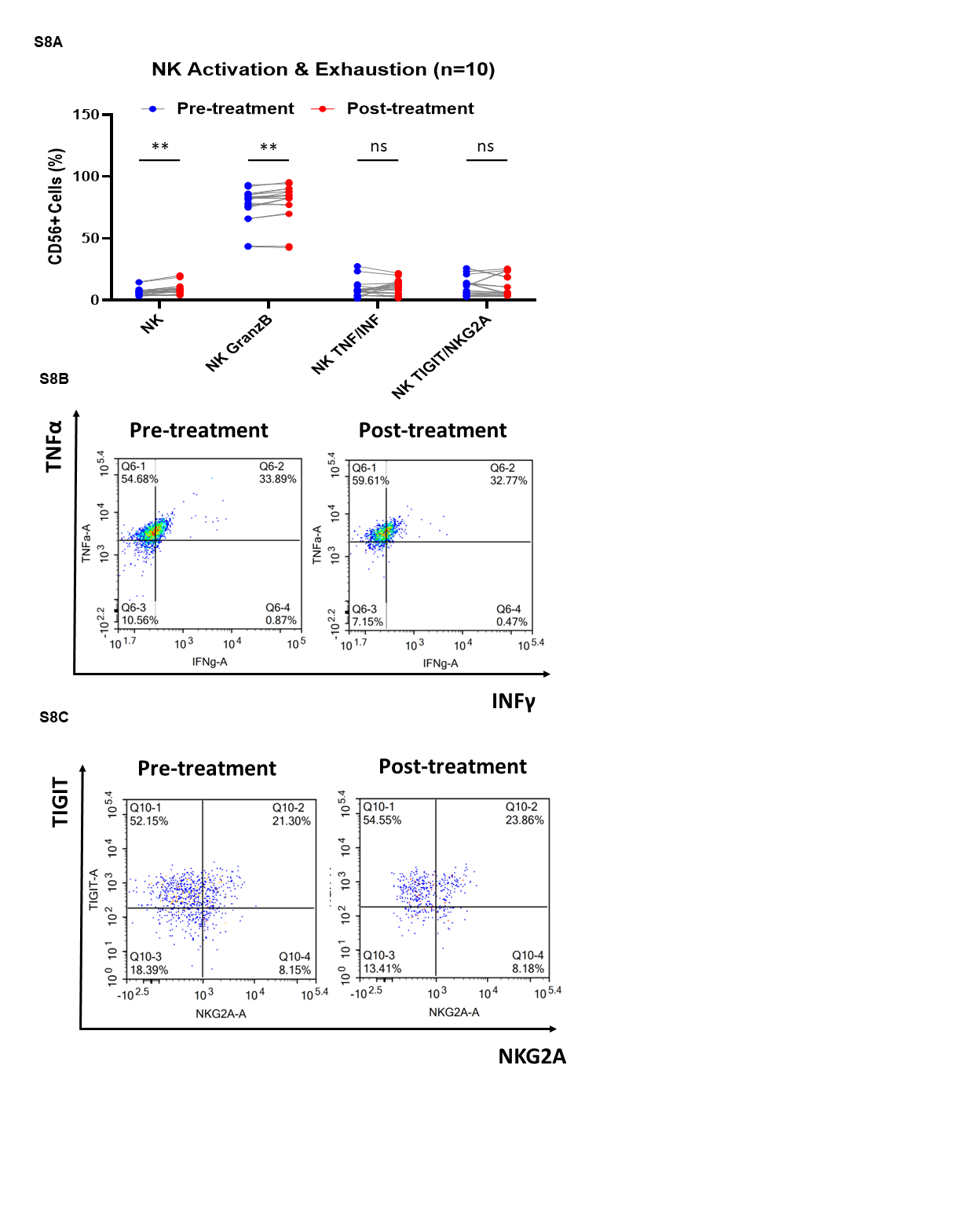


**Fig. S9: Seurat analysis identifying various immune cell populations, monocyte and neutrophil subclusters, in the discovery and validation datasets.** The heatmaps present the top 10 marker genes for **A.** each of the major immune cell types (neutrophils, monocytes, CD4+ T cells, CD8+ T cells, NK cells, B cells, and dendritic cells) in blood samples; **B.** monocyte subclusters (M1~M3), and **C.** neutrophile subclusters (N1~N4) from Discovery dataset (n=4 patients, 8 samples) and Validation dataset (n=4 patients, 8 samples).


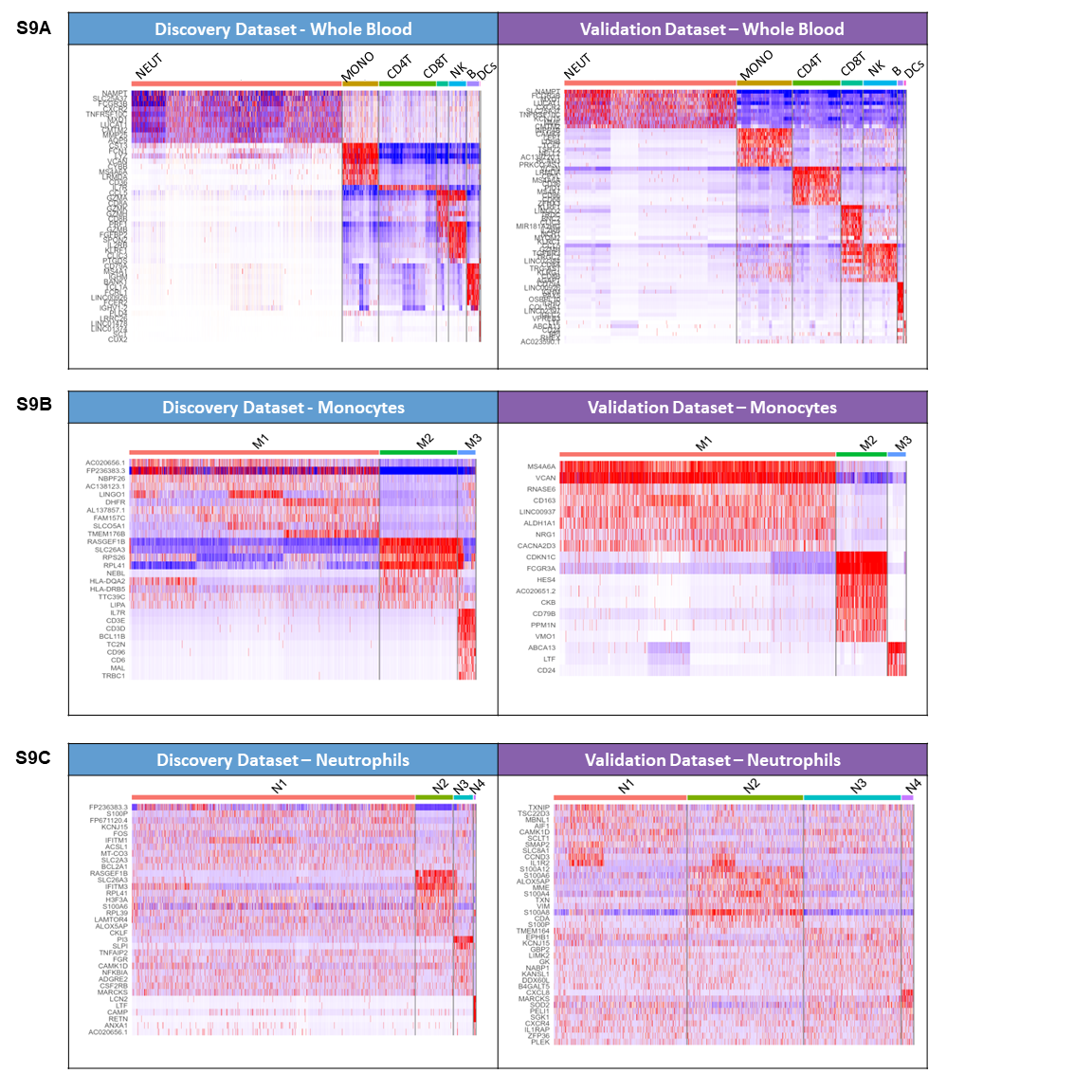


**Fig. S10: WBM Intake by PCa Patients Influences Functional Gene Expression in PMN-MDSC Subclusters. A.** The heatmaps present the top 10 marker genes for PMN-MDSCs subclusters (PMN-M1 to M4) in discovery and validation datasets. **B.** The violin plots demonstrate the expression levels of genes related to PMN-MDSCs differentiation and immunosuppressive functions. Genes include *ARG1, PTGS2, CXCR2, TGFB1, IL1B, MMP9, STAT3,* and *IRF1*, which are in each of the identified PMN-MDSC clusters from Discovery dataset (n=4 patients, 8 samples) and Validation dataset (n=4 patients, 8 samples).


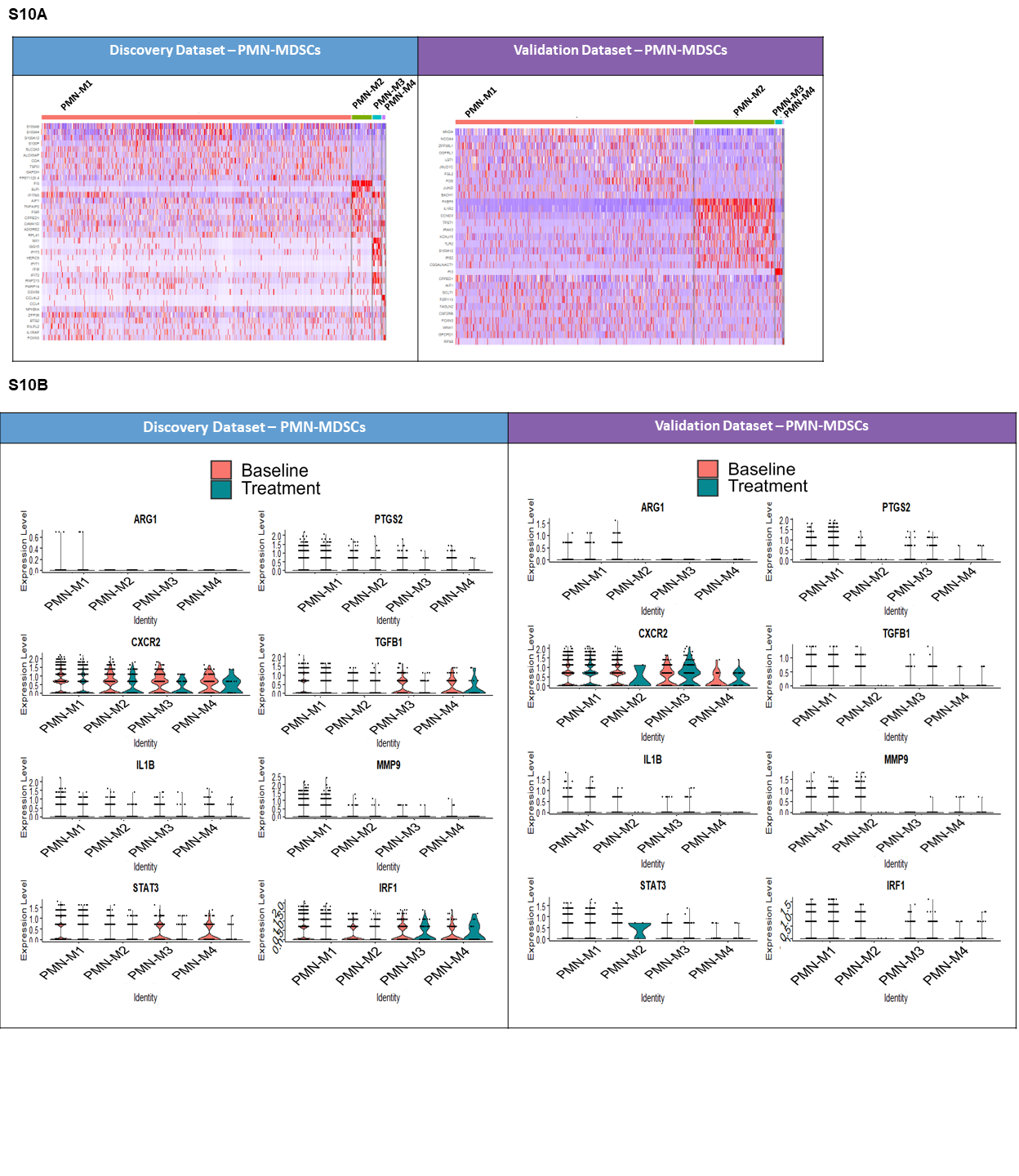


**Fig. S11: WBM Intake by PCa Patients Affects Functional Gene Expression in T/NK Cell Subclusters. A.** The violin plots demonstrate the expression levels of genes linked to T cell activation and exhaustion. Genes such as *PDCD1, CTLA4, HAVCR2, LAG3, TIGIT, IFNG, TNF,* and *GZMB* are depicted in CD4^+^ T cells, CD8^+^ T cells, and other T cells. **B**. The violin plots demonstrate the expression levels of genes linked to NK cell activation and exhaustion. Genes like *TIGIT, KLRC1, GZMB, PRF1, TNF,* and *IFNG* are highlighted in NK cells, NK proliferating cells, and NK CD56 bright cells from Discovery dataset (n=4 patients, 8 samples) and Validation dataset (n=4 patients, 8 samples).


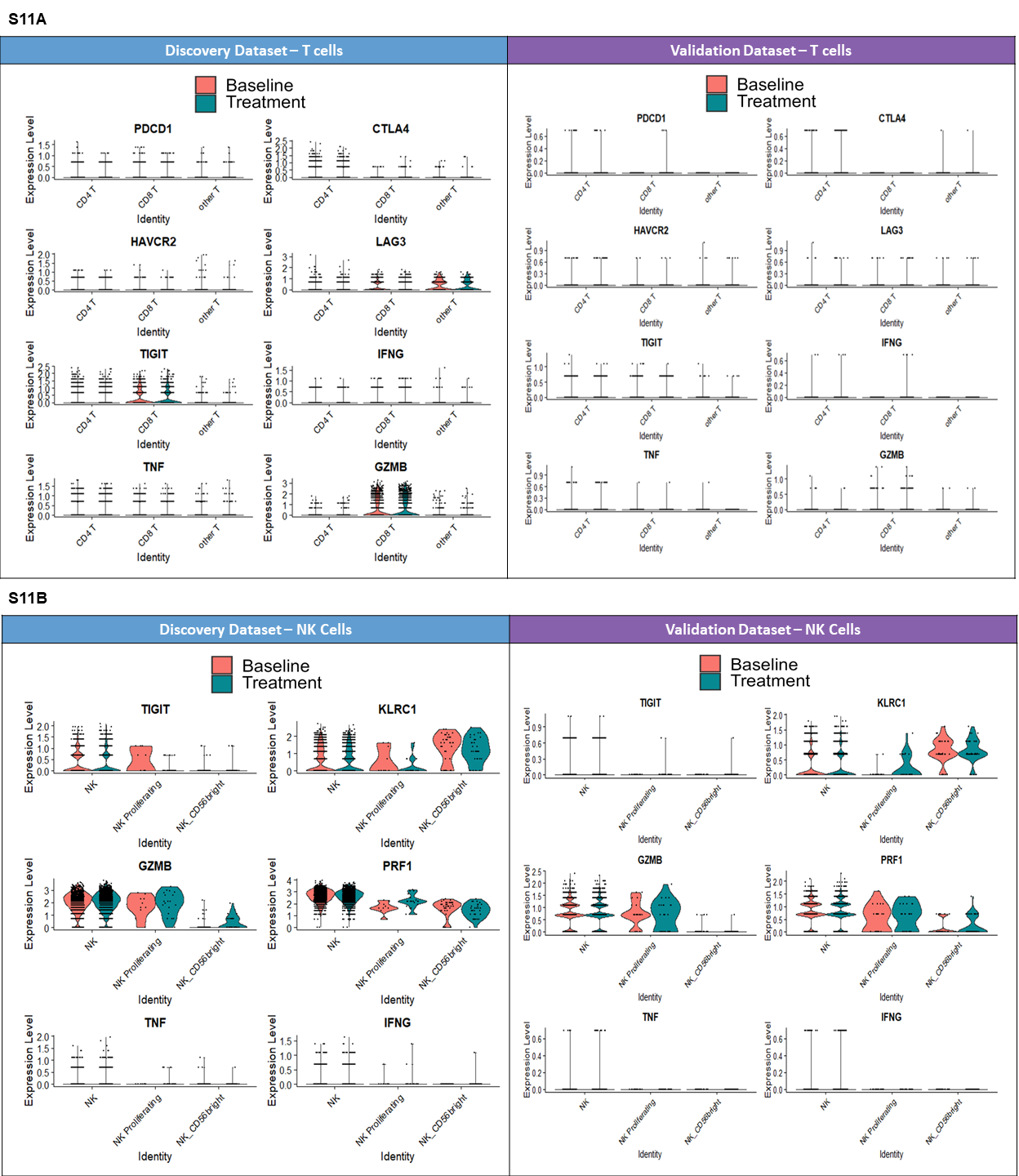


**Fig. S12: Bliss Independence Calculation for Evaluating the Drug Combination of WBM and Anti-PD-1 Treatment.** This figure presents a bar plot illustrating the percentage of average tumor size in each treatment group (n=5, A = WBM + Isotype, B = PBS + anti-PD-1, A + B = WBM + anti-PD-1) and the control group (n=5, PBS + Isotype). Additionally, the Bliss Independence calculation was performed to assess the treatment impact of the combination group (A + B).


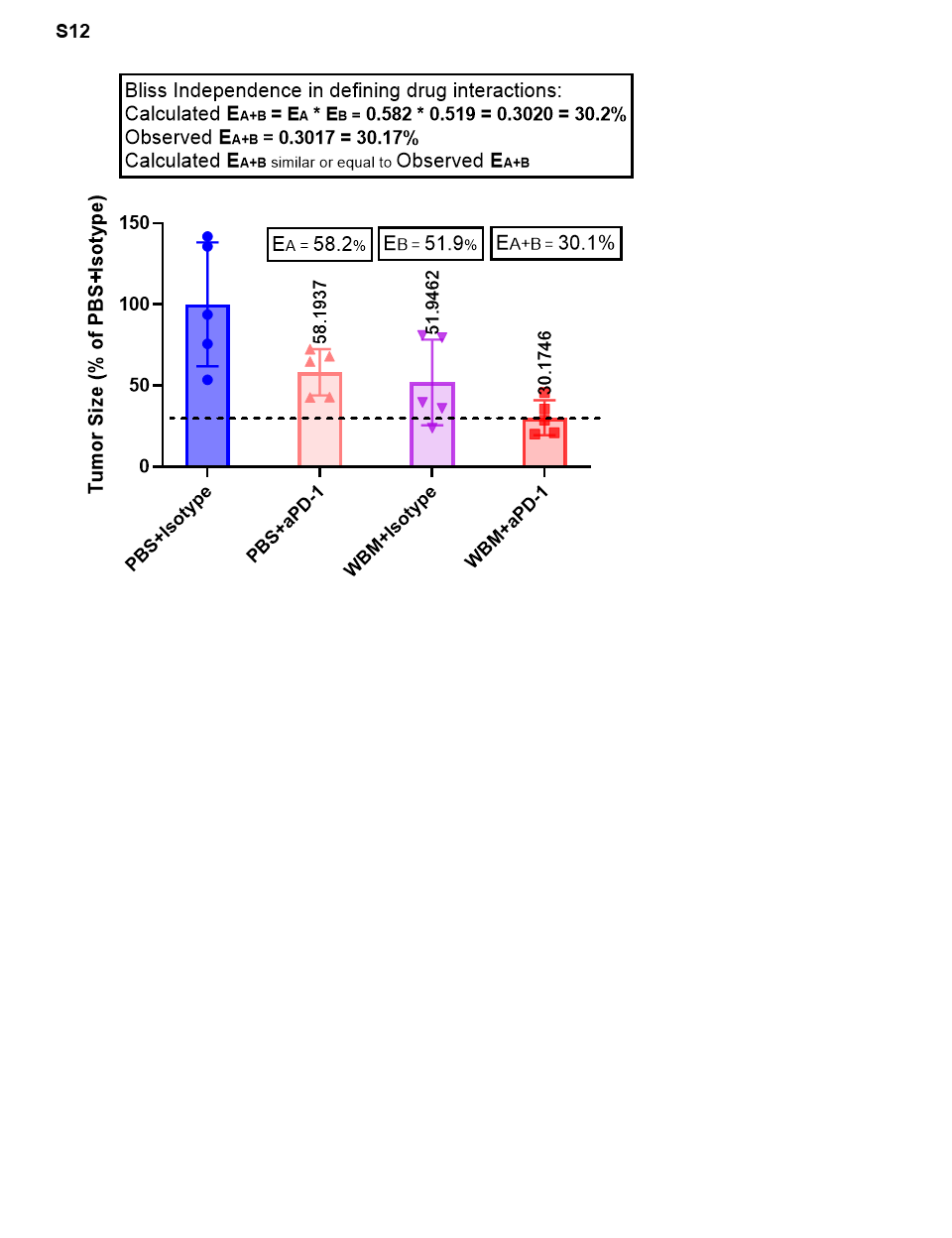


**Table S1. List of mRNA genes with differential expression in mouse PCa xenograft tumors treated with WBM, as detected by the NanoString PanCancer IO 360^TM^ Panel.**

| Gene | Log2 fold | p-value | Function |
| --- | --- | --- | --- |
| *Pdcd1* | 2.19 | 0.00413 | CD molecules, Humoral, T Cell Functions |
| *Cd3d* | 2.01 | 0.00586 | CD molecules, T Cell Functions |
| *Card11* | 1.79 | 0.00232 | B Cell Functions, Cytokines & Receptors, Interleukins, T Cell Functions |
| *Cd22* | 1.74 | 0.00453 | Adhesion, B Cell Functions, CD molecules |
| *H2-Eb1* | 1.74 | 0.00983 | Antigen Processing, Interleukins, MHC |
| *Tnfsf13b* | 1.61 | 0.00462 | B Cell Functions, CD molecules, Cytokines & Receptors, T Cell Functions, TNF Superfamily |
| *Psmb9* | 1.58 | 0.00832 | Antigen Processing |
| *Il6ra* | 1.45 | 0.00806 | CD molecules, Cytokines & Receptors, Interleukins |
| *Cxcr3* | 1.44 | 0.00203 | Adaptive, CD molecules, Cytokines & Receptors, Innate, T Cell Functions |
| *Cd3g* | 1.4 | 0.00639 | CD molecules, T Cell Functions, Transporter Functions |
| *Relb* | 1.4 | 0.00853 | Antigen Processing, Dendritic Cell Functions, T Cell Functions |
| *H2-T23* | 1.39 | 0.00741 | Antigen Processing, MHC, T Cell Functions |
| *Tnfrsf1b* | 1.36 | 0.00338 | CD molecules, Inflammation, TNF Superfamily |
| *Lck* | 1.36 | 0.00969 | B Cell Functions, T Cell Functions |
| *Abcg1* | 1.3 | 0.00620 | Innate, Transporter Functions |
| *Klra6* | 1.25 | 0.00393 | Adhesion, NK Cell Functions |
| *H2-K1* | 1.23 | 0.00907 | Antigen Processing, MHC, T Cell Functions |
| *Mif* | 1.21 | 0.00551 | B Cell Functions, Cytokines & Receptors, Inflammation, Innate, Transporter Functions |
| *Tnfrsf14* | 1.19 | 0.00894 | CD molecules, T Cell Functions, TNF Superfamily |
| *Itgae* | 1.18 | 0.0022 | Adhesion, CD molecules |
| *Tlr5* | 1.15 | 0.00123 | Inflammation, Innate, Interleukins |
| *Ikzf2* | 1.11 | 0.00656 | T Cell Functions |
| *Klra4* | 1.1 | 0.00103 | Adhesion, NK Cell Functions |
| *Itgb2* | 1.09 | 0.00513 | CD molecules, Humoral, Inflammation, Leukocyte Functions, NK Cell Functions, T Cell Functions, Transporter Functions |
| *Il1rl1* | 1.09 | 0.00786 | Cytokines & Receptors, Inflammation, Innate, Interleukins, Macrophage Functions, Transporter Functions |
| *C3ar1* | 1.09 | 0.00810 | Adaptive, Complement Pathway, Inflammation, Macrophage Functions |
| *Il2rg* | 1.07 | 0.00465 | B Cell Functions, CD molecules, Cytokines & Receptors, Interleukins, T Cell Functions |
| *Pou2f2* | 1.04 | 0.00847 | Humoral, T Cell Functions |
| *Cd84* | 1.03 | 0.00648 | Adhesion, CD molecules |
| *Nfkb1* | 1.03 | 0.00732 | Adaptive, Cytokines & Receptors, Humoral, Inflammation, Innate, Interleukins, Senescence, T Cell Functions |
| *Casp8* | 1.01 | 0.00261 | Innate, Macrophage Functions |
| *Cd47* | -1.2 | 0.00429 | CD molecules, Inflammation, T Cell Functions, Transporter Functions |
| *Clu* | -2 | 0.00581 | Innate |

The table includes a list of genes associated with the immune response of WBM treatment, which were identified through NanoString nCounter gene expression study. The input samples came from mouse PCa xenograft tumors from the control treatment group (PBS, n=3) and the WBM treatment group (WBM, n=3).

**Table S2. Biological processes related to the immunological function induced by WBM treatment in mouse PCa xenograft tumors, as detected by the NanoString PanCancer IO 360^TM^ Panel.**

|  | P value | DF | Regulated Genes |
| --- | --- | --- | --- |
| T-Cell Functions | 0.025939 | 2.728 | *Card11, Cd3d, Cd3g, Cd47, Cxcr3, H2-K1, H2-T23, Ikzf2, Il2rg, Itgb2, Lck, Nfkb1, Pdcd1, Pou2f2, Relb, Tnfrsf14, Tnfsf13b* |
| CD molecules | 0.022036 | 2.194 | *Cd22, Cd3d, Cd3g, Cd47, Cd84, Cxcr3, Il2rg, Il6ra, Itgae, Itgb2, Pdcd1, Tnfrsf1b, Tnfsf13b, Tnfrsf14* |
| Antigen Processing | 0.058163 | 2.129 | *H2-Eb1, H2-K1, H2-T23, Psmb9, Relb* |
| TNF Superfamily | 0.028798 | 1.913 | *Tnfrsf1b, Tnfsf13b, Tnfrsf14* |
| Cell Cycle | 0.028324 | 1.673 | *Runx3* |
| Interleukins | 0.010575 | 1.503 | *Card11, H2-Eb1, Il1rl1, Il2rg, Il6ra, Nfkb1, Tlr5* |
| Inflammation | 0.023311 | 1.455 | *C3ar1, Cd47, Il1rl1, Itgb2, Mif, Nfkb1, Tlr5, Tnfrsf1b* |
| Cytokines & Receptors | 0.017425 | 1.394 | *Card11, Cxcr3, Il1rl1, Il2rg, Il6ra, Mif, Nfkb1, Tnfsf13b* |
| Basic Cell Functions | 0.079008 | 1.341 | *Hcst* |
| Chemokines & Receptors | 0.030034 | 1.329 | *Card11, Cxcr3, Il1rl1, Il2rg, Il6ra, Mif, Nfkb1, Tnfsf13b* |
| Dendritic Cell Functions | 0.017131 | 1.26 | *Relb* |
| TLR | 0.014104 | 1.181 | *Tlr5, Tlr8, Irak2* |
| Adhesion | 0.028181 | 1.15 | *Cd22, Cd84, Itgae, Klra4, Klra6* |
| Interferon | 0.041824 | 1.113 | *Cd3e, Nlrc5* |
| Humoral | 0.018427 | 1.084 | *Itgb2, Nfkb1, Pdcd1, Pou2f2* |
| Microglial Functions | 0.038664 | 1.055 | *Nod2* |
| Adaptive | 0.021747 | 0.9945 | *C3ar1, Cxcr3, Nfkb1* |
| Apoptosis | 0.029222 | 0.9748 | *Pdcd1* |
| Macrophage Functions | 0.012514 | 0.9053 | *Casp8, C3ar1, Il1rl1* |
| Innate | 0.037276 | 0.8933 | *Abcg1, Casp8, Clu, Cxcr3, Il1rl1, Mif, Nfkb1, Tlr5* |
| Cancer Progression | 0.067311 | 0.825 | *C3ar1, Casp8, Ccl5* |
| Pathogen Response | 0.03839 | 0.7975 | *Il1b, Tlr8, Tnf* |
| Complement Pathway | 0.052597 | 0.7594 | *C3ar1* |
| NK Cell Functions | 0.022268 | 0.674 | *Itgb2, Klra4, Klra6* |
| Senescence | 0.063674 | 0.5929 | *Nfkb1* |
| Leukocyte Functions | 0.026689 | 0.5911 | *Itgb2* |
| MHC | 0.053141 | 0.5506 | *H2-Eb1, H2-K1, H2-T23* |
| B-Cell Functions | 0.024461 | 0.4973 | *Card11, Cd22, Il2rg, Lck, Mif, Tnfsf13b* |
| Transporter Functions | 0.039784 | 0.4766 | *Abcg1, Cd3g, Cd47, Il1rl1, Itgb2, Mif* |

Nanostring’s nCounter^®^ PanCancer IO360^TM^ panel was used to generate pathway scores for 29 different biological pathways associated with immune response genes. The input samples were mice prostate tumors collected from the control treatment (PBS, n=3) group and the WBM (n=3) treatment group. Statistical analysis was performed by comparing the data from the control and the WBM treatment groups. DF, differential folds; TLR, Toll-like receptors; MHC, Major histocompatibility complex.

**Table S3. List of discovery and validation samples involved in single immune cells gene expression profiling study.**

| Discovery Cohort (n=4) | Discovery Dataset (GSE266985) | |
| --- | --- | --- |
|  | Baseline (cell number) | 3-month Treatment (cell number) |
| Patient 1 | 3,077 | 3,754 |
| Patient 2 | 1,925 | 2,652 |
| Patient 3 | 8,441 | 3,617 |
| Patient 4 | 5,539 | 2,645 |
| Sum | 18,982 | 12,668 |
|  | | |
| Validation Cohort (n=4) | **Validation Dataset (GSE275574)** | |
|  | Baseline (cell number) | 3-month Treatment (cell number) |
| Patient 5 | 8,808 | 11,875 |
| Patient 6 | 4,642 | 7,281 |
| Patient 7 | 9,541 | 8,415 |
| Patient 8 | 12,390 | 10,312 |
| Sum | 35,378 | 37,883 |

**Table S4. List of chemicals and mouse antibodies**. Mouse lymphoid and myeloid cells were identified by staining with the following panel of blocking solution and antibodies.

| Antibodies/ Chemicals Used | Clone | Isotype | Cat. no. | Source |
| --- | --- | --- | --- | --- |
| TruStain FCX™ PLUS (anti-mouse CD16/32) | S17011E | Rat IgG2b, κ | 156604 | BioLegend |
| FITC anti-mouse CD3ε | 500A2 | Syrian Hamster IgG | 152304 | BioLegend |
| PE anti-mouse CD4 | GK1.5 | Rat IgG2b, κ | 100408 | BioLegend |
| APC anti-mouse CD8a | 53-6.7 | Rat IgG2a, κ | 100712 | BioLegend |
| PE anti-mouse NK-1.1 | PK136 | Mouse IgG2a, κ | 108708 | BioLegend |
| APC anti-mouse CD49b (pan-NK cells) | DX5 | Rat IgM, κ | 108910 | BioLegend |
| PerCP/Cyanine5.5 anti-mouse IFN-γ | XMG1.2 | Rat IgG1, κ | 505822 | BioLegend |
| PerCP/Cyanine5.5 anti-human/mouse Granzyme B Recombinant | QA16A02 | Mouse IgG1, κ | 372212 | BioLegend |
| PerCP/Cyanine5.5 anti-mouse CD279 (PD-1) | RMP1-30 | Rat IgG2b, κ | 109120 | BioLegend |
| FITC anti-mouse CD45 | 30-F11 | Rat IgG2b, κ | 103108 | BioLegend |
| Anti-Mo/Rt FOXP3, eBioscience™ PerCP-Cyanine5.5 | FKJ-16s | Rat IgG2a, κ | 45-5773-82 | Invitrogen |
| APC anti-mouse I-A/I-E | M5/114.15.2 | Rat IgG2b, κ | 107614 | BioLegend |
| PerCP/Cyanine5.5 anti-human/mouse CD11b | M1/70 | Rat IgG2b, κ | 101228 | BioLegend |
| PE anti-mouse Ly-6G | 1A8 | Rat IgG2a, κ | 127608 | BioLegend |
| APC anti-mouse Ly-6C | HK1.4 | Rat IgG2C, κ | 128016 | BioLegend |
| APC anti-mouse F4/80 Recombinant | QA17A29 | Mouse IgG1, κ | 157306 | BioLegend |
| PE anti-mouse CD274 (B7-H1, PD-L1) | 10F.9G2 | Rat IgG2b, κ | 124308 | BioLegend |
| PE anti-mouse CD11c | N418 | Armenian Hamster IgG | 117308 | BioLegend |
| PerCP/Cyanine5.5 anti-human Arginase I | 14D2C43 | Mouse IgG2b, κ | 369710 | BioLegend |
| iNOS Monoclonal Antibody (CXNFT), Alexa Fluor™ 488, eBioscience™ | CXNFT | Rat IgG2a, κ | 53-5920-82 | Invitrogen |
| monoclonal EPR25A -Isotope |  | Rabbit IgG | ab172730 | abCam |

**Table S5. List of chemicals and human antibodie**s. Human lymphoid and myeloid cells were identified by staining with the following panel of blocking solution and antibodies.

| Antibodies/ Chemicals Used | Clone | Isotype | Cat. no. | Source |
| --- | --- | --- | --- | --- |
| Human TruStain FCX™ |  |  | 422302 | BioLegend |
| eBioscience™ Cell Stimulation Cocktail (500X) |  |  | 00-4970-93 | Invitrogen |
| PE anti-human CD80 | W17149D | Rat IgG2a, κ | 375410 | BioLegend |
| FITC anti-human IFN-γ | B27 | Mouse IgG1, κ | 506504 | BioLegend |
| Brilliant Violet 421™ anti-human CD274 (B7-H1, PD-L1) | 29E.2A3 | Mouse IgG2b, κ | 329714 | BioLegend |
| BD OptiBuild™ BV510 Mouse Anti-Human NKG2A (CD159a) | 131411 | Mouse IgG2a, κ | 747922 | BD Biosciences |
| PE/Cyanine7 anti-human CD366 (Tim-3) | F38-2E2 | Mouse IgG1, κ | 345014 | BioLegend |
| Brilliant Violet 605™ anti-human CD152 (CTLA-4) | BNI3 | Mouse IgG2a, κ | 369610 | BioLegend |
| PE anti-human TIGIT (VSTM3) | A15153G | Mouse IgG2a, κ | 372704 | BioLegend |
| BD Horizon™ PE-CF594 Mouse Anti-Human CD3 | UCHT1 | Mouse BALB/c IgG1, κ | 562280 | BD Biosciences |
| APC anti-human CD8 | SK1 | Mouse IgG1, κ | 344722 | BioLegend |
| PE/Cyanine7 anti-human/mouse Granzyme B Recombinant | QA16A02 | Mouse IgG1, κ | 372214 | BioLegend |
| Alexa Fluor® 700 anti-human CD56 (NCAM) | HCD56 | Mouse IgG1, κ | 318316 | BioLegend |
| Brilliant Violet 421™ anti-human CD4 | RPA-T4 | Mouse IgG1, κ | 300532 | BioLegend |
| FITC anti-human CD279 (PD-1) | A17188B | Mouse IgG2b, κ | 621612 | BioLegend |
| BD Horizon™ PE-CF594 Mouse Anti-Human CD86 | 2331 (FUN-1) | Mouse BALB/c IgG1, κ | 562390 | BD Biosciences |
| PerCP/Cyanine5.5 anti-human Arginase I | 14D2C43 | Mouse IgG2b, κ | 369710 | BioLegend |
| APC anti-human CD206 (MMR) | 15-2 | Mouse IgG1, κ | 321110 | BioLegend |
| APC anti-human CD15 (SSEA-1) | HI98 | Mouse IgM, κ | 301908 | BioLegend |
| PE anti-human CD14 | M5E2 | Mouse IgG2a, κ | 301806 | BioLegend |
| PE/Cyanine7 anti-human CD11c | 3.9 | Mouse IgG1, κ | 301607 | BioLegend |
| Per-CP/Cyanine5.5 anti-human HLA-DR | L243 | Mouse IgG2a, κ | 307630 | BioLegend |
| PE anti-human TNF-α | MAb11 | Mouse IgG1, κ | 502909 | BioLegend |
| FITC anti-mouse/human CD11b | M1/70 | Rat IgG2b, κ | 101206 | BioLegend |
| Arginase 1 Monoclonal Antibody (A1exF5), APC, eBioscience™ | A1exF5 | Rat IgG2a, κ | 17-3697-82 | Invitrogen |
| Anti-Hu/Mo Arginase-1, eBioscience™ PE | A1exF5 | Rat IgG2a, κ | 12-3697-82 | Invitrogen |

**Table S6. List of mouse primer sequences used for qRT-PCR.**

| Genes | Sequences (Forward) | | Sequences (Reverse) |
| --- | --- | --- | --- |
| Ly6C | | 5'- GCA GTG CTA CGA GTG CTA TGG -3' | 5' - ACT GAC GGG TCT TTA GTT TCC TT -3' |
| Ly6G | | 5'- GAC TTC CTG CAA CAC AAC TAC C -3' | 5'- ACA GCA TTA CCA GTG ATC TCA GT -3' |
| STAT3 | | 5'- CAA TAC CAT TGA CCT GCC GAT -3' | 5'- GAG CGA CTC AAA CTG CCC T -3' |
| S100A8 | | 5'- AAA TCA CCA TGC CCT CTA CAA G -3' | 5'- CCC ACT TTT ATC ACC ATC GCA A -3' |
| S100A9 | | 5'- ATA CTC TAG GAA GGA AGG ACA CC -3' | 5'- TCC ATG ATG TCA TTT ATG AGG GC -3' |
| ARG1 | | 5'- CTC CAA GCC AAA GTC CTT AGA G -3' | 5'- AGG AGC TGT CAT TAG GGA CAT C -3' |
| ARG2 | | 5'- TCC TCC ACG GGC AAA TTC C -3' | 5'- GCT GGA CCA TAT TCC ACT CCT A -3' |
| NOS2 | | 5'- GTT CTC AGC CCA ACA ATA CAA GA -3' | 5'- GTG GAC GGG TCG ATG TCA C -3' |
| TGFβ | | 5'- CTC CCG TGG CTT CTA GTG C -3' | 5'- GCC TTA GTT TGG ACA GGA TCT G -3' |
| 1L-1β | | 5'- GCA ACT GTT CCT GAA CTC AAC T -3' | 5'- ATC TTT TGG GGT CCG TCA ACT -3' |
| IL-6 | | 5'- TAG TCC TTC CTA CCC CAA TTT CC -3' | 5'- TTG GTC CTT AGC CAC TCC TTC -3' |
